# Measuring Coverage in MNCH: Testing the Validity of Women's Self-Report of Key Maternal and Newborn Health Interventions during the Peripartum Period in Mozambique

**DOI:** 10.1371/journal.pone.0060694

**Published:** 2013-05-07

**Authors:** Cynthia K. Stanton, Barbara Rawlins, Mary Drake, Matias dos Anjos, David Cantor, Lidia Chongo, Leonardo Chavane, Maria da Luz Vaz, Jim Ricca

**Affiliations:** 1 Johns Hopkins Bloomberg School of Public Health, Baltimore, Maryland, United States of America; 2 Maternal and Child Health Integrated Program, Washington, DC, United States of America; 3 Maternal and Child Health Integrated Program, Maputo, Mozambique; 4 Mozambique Ministry of Health, Maputo, Mozambique; Kings College London, United Kingdom, in consultation with Carla AbouZahr, independent consultant, health statistics and policy.

## Abstract

**Background:**

As low-income countries strive to meet targets for Millennium Development Goals 4 and 5, there is growing need to track coverage and quality of high-impact peripartum interventions. At present, nationally representative household surveys conducted in low-income settings primarily measure contact with the health system, shedding little light on content or quality of care. The objective of this study is to validate the ability of women in Mozambique to report on facility-based care they and their newborns received during labor and one hour postpartum.

**Methods and Findings:**

The study involved household interviews with women in Mozambique whose births were observed eight to ten months previously as part of a survey of the quality of maternal and newborn care at government health facilities. Of 487 women whose births were observed and who agreed to a follow-up interview, 304 were interviewed (62.4%). The validity of 34 indicators was tested using two measures: area under receiver operator characteristic curve (AUC) and inflation factor (IF); 27 indicators had sufficient numbers for robust analysis, of which four met acceptability criteria for both (AUC >0.6 and 0.75<IF<1.25). Two of these indicators are considered high demand and are recommended for incorporation into international survey programs: presence of a support person during labor/delivery and placement of the newborn skin to skin against the mother. Nine indicators met acceptability criteria for one of the validity measures. All 13 indicators are recommended for use in in-depth maternal/newborn health surveys.

**Conclusions:**

Women are able to report on some aspects of peripartum care. Larger studies may be able to validate some indicators that this study could not assess due to the sample size. Future qualitative research may assist in improving question formulation for some indicators. Studies of similar design in other low-income settings are needed to confirm these results.

*This paper is part of the* PLOS Medicine *"Measuring Coverage in MNCH" Collection.*

## Introduction

As low-income countries strive to meet their targets for Millennium Development Goals 4 and 5, there is an urgent need to increase use and quality of maternal, newborn, and child health (MNCH) care services. To better monitor global trends, there is a strong demand to improve MNCH coverage indicators, spurred mainly by efforts in response to the Commission on Information and Accountability for Women's and Children's Health [1] and the Countdown to 2015 initiative [2]. Such indicators are also critical at the national level to provide actionable information regarding the adequacy of the content and quality of MNCH care to achieve desired health outcomes.

Presently, nationally representative household surveys conducted in low-income settings, such as the Demographic and Health Surveys (DHS) and the Multiple Indicator Cluster Surveys (MICS) track few indicators that measure care during the intrapartum and immediate postpartum periods. Typically, these are limited to: location of birth, qualification of birth attendant, and cesarean section. The first two quantify contact with the health system, but provide no information on content of care. The validity of even these commonly reported survey indicators has not been assessed. However, national and international monitoring relies heavily on DHS and MICS survey data due to the inadequate state of routine health information system data in many low-income countries resulting from incomplete and irregular reporting, errors in manual calculation, incomplete or irregular data transmission from lower to higher levels of the health system, and compilation of subsets of indicators at irregular intervals by multiple divisions within the ministries of health.

A review of published literature from the past 30 years yielded multiple studies validating women's report of MNCH-related outcomes and health care in high-resource settings [3]–[20] but few from low-resource settings [21]–[26]. All studies used clinical records as the reference standard. The six studies from low-resource countries [21]–[26] all validated women's self-reports of obstetric complications comparing follow-up interviews with women who delivered in hospitals against clinical records. In the *PLOS Medicine* “Measuring Coverage in MNCH” Collection, of which this paper is a part, two other articles address indicators for care in the peripartum period [27], [28].

The objective of this study was to assess the validity of women's self-reports of selected health facility–based, peripartum MNCH interventions in Mozambique in two complementary ways: (1) via calculation of sensitivity and specificity, and area under receiver operator characteristic curve (AUC); and (2) estimation of the inflation factor (IF), which is the ratio of the prevalence of these interventions that would be obtained from a population-based survey, given the sensitivity and specificity from this study, and the indicator's true prevalence.

Mozambique was chosen for this study because it is a priority country for the Countdown to 2015 initiative and is typical of the other 74 countries which represent 95% of maternal and child deaths globally. Mozambique is similar particularly to other sub-Saharan African countries: maternal mortality ratio is high (500 maternal deaths per 100,000 live births) [29], the institutional birth rate is 54% [29], there are few births in private or non-governmental facilities, approximately 85% of women of reproductive age have achieved at most primary school education, and just over one third (38%) of its population resides in an urban area [30].

## Methods

### Ethical Review

This study was approved by the Mozambique National Bioethics Committee and the Institutional Review Board of the Johns Hopkins Bloomberg School of Public Health.

### Study Design

The study involved face-to-face interviews with women in Mozambique whose births were observed and documented as part of a government health facility survey of the Quality of MNCH Care (referred to subsequently as Quality of Care study) which was conducted from September to November 2011; results are available elsewhere [31]. Eight to ten months later, study participants were interviewed in their homes regarding the care they received during labor, delivery, and up to approximately one hour following birth. Data from the follow-up interviews were compared against data from the observations, which served as the reference standard. Because this validation study focused on facility-based care, the sample consisted of women who delivered in a health facility.

Indicators included in this study were selected based on three criteria: (1) evidence-based interventions during the peripartum period identified in the WHO Integrated Management of Pregnancy and Childbirth (IMPAC) manuals [32], [33]; (2) important elements of the Mozambique humanization of birth program; and (3) events considered feasible for a woman to report on (e.g., inquiries or physical interventions by birth attendants versus the conduct of laboratory tests). Where questions of interest already exist in the DHS and/or MICS surveys, we tested the same or similar formulations of the questions.

The list of indicators selected for validity testing is presented in Table 1. Given constraints on the length of large-scale survey questionnaires, this list differentiates between high-demand indicators that are potential candidates for inclusion into the DHS/MICS questionnaires and indicators appropriate for more in-depth surveys of maternal and newborn health. The table also identifies indicators as beneficial or harmful. To note, some indicators are neither. These indicators represent two types of practice: (1) interventions that would require subjective information as to whether the intervention was medically indicated; for example, augmentation of labor or cesarean section; such judgments were beyond the scope of this study; and (2) indicators that are by their nature neither harmful or beneficial, such as choosing to deliver at a hospital versus a health center.

**Table 1:** List of 34 indicators for which validation was attempted in this study.

Question Already Exists in DHS/MICS Surveys	High-Demand Indicators Potentially Suggested for Use in DHS/MICS-Type Surveys	Indicator of Beneficial or Harmful Care	Indicator
			**Initial Assessment of a Woman in Labor**
	Yes	Beneficial	Percent of women asked about their HIV status
		Beneficial	AMONG WOMEN WITH UNKNOWN HIV STATUS: Percent of women offered HIV test
	Yes	Beneficial	Percent of women who had their blood pressure taken
	Yes	Beneficial	Percent of women who were asked for a urine sample upon arrival at the health facility
			**Intra-partum Care**
		Beneficial	Percent of women who were encouraged to have a companion present during labor/delivery
	Yes	Beneficial	Percent of women who had a companion present during labor or delivery
		Beneficial	Percent of women who were encouraged to ambulate or move around during labor
		Beneficial	Percent of women who were draped for privacy during labor
		Harmful	Percent of women who were slapped, physically mistreated
		Harmful	Percent of women who were shouted at or otherwise verbally mistreated
		Neither	Percent of women who had their labor augmented with injection
		Neither	Percent of births with cephalic presentation
		Neither	Percent of women who had more than one health care provider assisting during birth
Yes		Neither	Percent of women who delivered in a hospital (versus a health center)
Yes		Neither	Percent of women who delivered by cesarean section
		Neither	Percent of women with an instrumental birth (forceps, vacuum extraction)
		Neither	Percent of women who delivered on their backs (lithotomy position)
		Neither	Percent of women who received an episiotomy
			**Immediate Postpartum/Postnatal Care**
	Yes	Beneficial	*Element of postpartum hemorrhage prevention:* Percent of women who received a uterotonic within 3 (a few) minutes after birth of the baby
	Yes	Beneficial	*Element of postpartum hemorrhage prevention:* Percent of women who received controlled cord traction
	Yes	Beneficial	*Element of postpartum hemorrhage prevention:* Percent of women who received fundal massage after delivery of the placenta
	Yes	Beneficial	*Composite indicator of active management of the third stage of labor:* Percent of women who received uterotonic within a few minutes after birth of baby, controlled cord traction, AND fundal massage after delivery of placenta
		Harmful	Percent of women who received fundal pressure before birth of baby
		Harmful	Percent of women for whom the birth attendant manually explored uterus after birth of baby
	Yes	Beneficial	*Element of thermal care for the newborn:* Percent of newborns^a^ who were immediately dried
	Yes	Beneficial	*Element of thermal care for the newborn:* Percent of newborns^a^ placed skin to skin against the mother's chest
	Yes	Beneficial	*Element of thermal care for the newborn:* AMONG NEWBORNS PLACED SKIN TO SKIN: Percent of newborns^a^ placed skin to skin on mother and covered with a cloth
	Yes	Beneficial	*Element of thermal care for the newborn:* AMONG NEWBORNS NOT PLACED SKIN TO SKIN: Percent of newborns^a^ wrapped in a towel/cloth
	Yes	Beneficial	*Composite indicator 1 of thermal care for the newborn:* AMONG NEWBORNS NOT PLACED SKIN TO SKIN: Percent of newborns^a^ immediately dried with a towel and wrapped with a cloth
	Yes	Beneficial	*Composite indicator 2 of thermal care for the newborn:* Percent of newborns^a^ immediately dried with a towel, placed skin to skin on mother and covered with a cloth
Yes		Beneficial	Percent of newborns^a^ for whom breastfeeding was initiated within one hour of birth
		Harmful	Percent of newborns^a^ held upside down
		Harmful	Percent of newborns^a^ slapped
		Harmful	Percent of newborns^a^ bathed within one hour (i.e., bathing not delayed)

a Any indicator referring to newborns or stillbirths refers to the second twin in cases of multiple birth.

High-demand, evidence-based intervention indicators that were identified as potential candidates for inclusion in MICS/DHS surveys include some based on one question: HIV status checked, blood pressure measurement, urine testing (interventions performed during initial assessment of a woman in labor), presence of support person during labor or delivery, together with indicators related to newborn thermal care and active management of the third stage of labor. Three composite indicators (based on two or more questions, as defined in Table 1) were identified: thermal care for the newborn defined in two ways: and active management of the third stage of labor. In one of the papers in this Collection, Moran et al. also place importance on two of the indicators above, endorsing the use of an indicator on immediate drying of the newborn and recommending testing the skin to skin indicator [28].

### Sample Size

Mozambique has a total of 650 public maternity units. In the Quality of Care observational Study [31], 46 government health facilities (20 hospitals and 26 health centers) with an average of at least three births per day were selected randomly from a group of 122 government health facilities included in a national initiative to improve the quality of maternity care. This larger group of health facilities is responsible for 50%–60% of institutional births nationally. At the time of the observations, government-sponsored training programs targeting evidence-based interventions during the peripartum period were underway in 19 of the 46 hospitals sampled. A total of 525 births were observed, including women whose births resulted in a stillbirth or early neonatal death, and some cases of women who eventually delivered via cesarean. There were no refusals in the Quality of Care Study [31].

For the validation study, the anticipated prevalence of study indicators ranged from 20% to 80%, given that some reflect preventive interventions and should be nearly universal, whereas others represent harmful practices and should rarely, if ever, be performed. Assumptions required for sample size calculation included: 50% prevalence for all indicators, 60% sensitivity (with precision at±7.0%), 70% specificity (or 30% [1 − specificity] with precision at±6.4%), and joint 90% confidence intervals [34] of 53%–67% for sensitivity and 23.6%–36.4% for (1 − specificity). Variation across facilities in the prevalence of indicators potentially influenced by in-service training programs was accounted for by basing sample size on 50% prevalence. Based on these assumptions, a sample of 400 follow-up interviews was required, increased to 480 to allow for 20% loss to follow-up and refusals.

### Data Collection

In the health facilities selected for the Quality of Care Study [31], all deliveries were observed between 7am and 11pm over two to three days (depending on the volume of births in the facility), with the following caveats: a single observer could not observe more than two deliveries simultaneously, and women admitted into the emergency ward or taken immediately for cesarean section were not observed. Births were observed by nurses trained to observe maternity care using a standard validated checklist. Following birth and before hospital discharge, study participants who granted permission to be interviewed at home several months later were asked for their address and phone number and detailed landmarks to assist the interviewer in locating the household.

All women who provided a phone number were called during the interim to remind them of the upcoming interview. Twelve interviewers were recruited, 11 of whom had previously served as interviewers for the 2011 Mozambique DHS survey. They did not have a medical background and were not the observers in the Quality of Care Study [31]. Interviewer training included review of biological and health care-related events during the peripartum period. Interviewers attempted to relocate study participants at home. Interviews were conducted in Portuguese, and data were entered directly into Android platform tablet computers running Mobile Data Studio data entry software, with data entry validation checks. Figures S1 and S2 provide the wording in Portuguese and English, respectively, of the each question supporting each indicator. Supervisors reviewed data for consistency and completeness before transmitting it electronically to a central web server connected to a Structured Query Language server database. The database was monitored on an ongoing basis. Data were exported to Stata Version 11 for analysis.

### Analysis

Two-by-two tables were constructed. If any cell had fewer than five cases, the analysis was not performed. Sensitivity and specificity were estimated for each indicator for which there was adequate information, with uncertainty represented by 95% confidence intervals assuming a binomial distribution. AUC quantifies the performance of a diagnostic test (in this case, a woman's response to a survey question compared against the health facility–based reference standard). The Receiver Operating Characteristic Curve is produced when the sensitivity of a test is plotted against 1 − specificity of the test. The area under the curve can then be estimated. The most common usage of this statistic is to estimate AUC for multiple cut-off points resulting from a single diagnostic test or to compare results from different diagnostic tests. An AUC of 1.0 represents a perfect diagnostic test, whereas an AUC of 0.50 represents a random guess. For the purposes of this study, AUC was estimated based on dichotomous variables and is used as a means of comparing overall validity across multiple indicators [35]. These traditional measures are used to assess validity at the individual level.

The prevalence of an indicator that would be obtained from a population-based survey, given the sensitivity and specificity of that indicator resulting from this study, was estimated using the equation below from Vecchio [36].



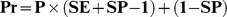



In this equation, Pr is the estimate of survey-based prevalence, P is the hypothetical “true” prevalence in the population, SE is sensitivity, and SP is specificity. Results regarding estimated population-based prevalence rates for selected indicators are expressed in this paper as the inflation factor, that is, as an over- or under-estimation factor relative to the true rate. Several other papers in this supplement have utilized the ratio of Test to Actual Positives (TAP ratio) [37], which is the mathematical equivalent of estimated prevalence as calculated by Vecchio and the IF. The IF is the ratio of the estimated survey-based prevalence to the true population prevalence, as measured in the Quality of Care Study [31]. The IF is used to represent population-based validity.

For those indicators for which the analysis could be conducted, we defined acceptability criteria for validation as an AUC>0.60 or an IF between 0.75 and 1.25. There is no consensus on acceptable levels of any of the validation measures used in this study. The cut-offs for AUC and IF are subjective and were selected prior to data collection following discussion among the investigators taking into account the complexity of the questions, the lengthy recall period, and the fact that women were answering questions about events during labor or the immediate postpartum period. Due to intense constraints to lengthening the DHS or MICS questionnaires, our acceptability criteria for new indicators warranting incorporation into international survey programs are stricter and include only those identified as “high demand” in Table 1 and those which meet *both* acceptability criteria. Thus, indicators recommended for the DHS and MICS surveys are those that showed accurate reporting at both the individual and population levels.

## Results

**Figure 1:**
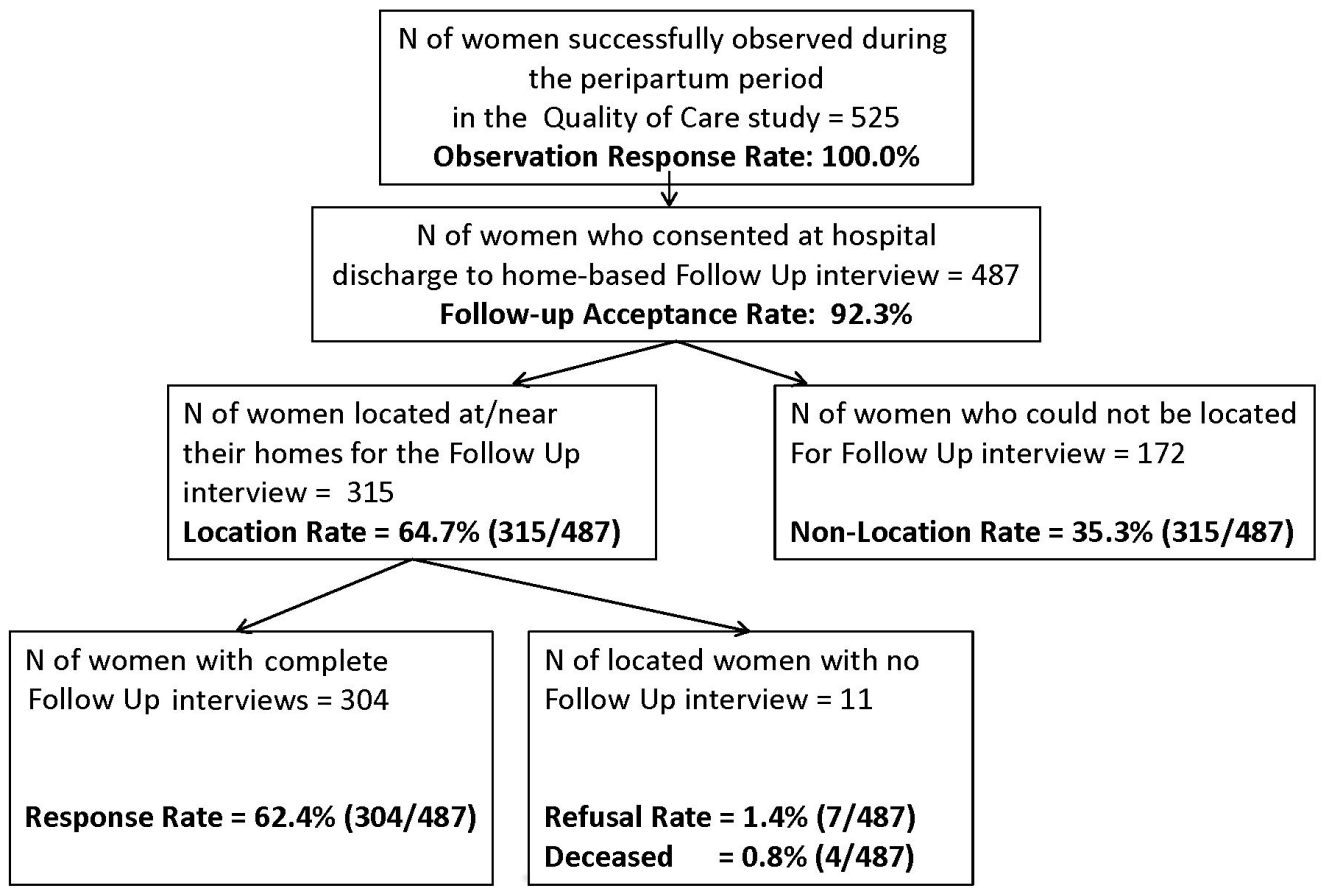
Response rates.

Of the 525 women observed in the Quality of Care Study [31], 92.3% consented to a follow-up interview (Figure 1). Interviewers were able to locate the households of 64.7% of women who provided consent, and among those, succeeded in interviewing 96.5%. Thus, loss to follow-up was substantially higher than assumed (37.6% versus 20%), and the final sample of 304 interviewed women fell short of the target sample of 400 women. Of note, 0.8% of the sample of women (n = 4) died in the interim between birth and the follow-up interview and 1.4% refused the interview.

Table 2 presents the distribution of background characteristics for respondents to the follow-up study, participants in the Quality of Care Study [31], and a nationally representative sample of respondents of the 2008 MICS survey who delivered in a health facility. All provinces in Mozambique are represented in the Quality of Care [31] and follow-up studies. However, in the follow-up study, Maputo City and Manica are over-represented and Inhambane is under-represented relative to nationally representative data. Women in the follow-up study were somewhat more educated, urban, and younger than MICS survey respondents.

**Table 2:** Background characteristics: Percent distribution of respondents in the follow-up study; Women whose deliveries were observed in the Quality of Care Study [31]; and Women who delivered in a health facility in the 2008 MICS (a nationally representative sample of women of reproductive age).

Socio-Demographic Characteristics	Follow-Up Survey (N = 304)	Mozambique Quality of Care Study (N = 525)	MICS 2008 (N = 3,011 Live Births in Two Years Preceding Survey)
**Age**			
13–19	17.8	26.1	(15–19) 17.6
20–24	33.9	29.9	29.3
25–29	22.4	21.0	23.0
30–34	15.1	14.1	16.3
35–39	8.6	7.0	10.5
40–44	2.0	0.8	2.5
45–49	0.3	0.0	0.8
Don't know/ missing	0.0	1.1	0.0
**Level of Education**			
None	12.8	Not available	23.8
Primary	55.6	Not available	62.2
Secondary or higher	31.6	Not available	13.4
Don't know/ missing	0.0	Not available	0.7
**Marital Status**			
Married/ Cohabitating	80.9	Not available	Not available
Single	12.5	Not available	Not available
Divorced/ Separated	6.6	Not available	Not available
**Area of Residence**			
Urban	54.0	55.0 a	39.9
Rural	43.1	44.0 a	60.1
Missing	3.0	1.0 a	0.0
**Region/Province**			
*North Region*			
Niassa	9.9	8.4	7.9
Cabo Delgado	6.3	3.8	7.9
Nampula	15.5	14.1	18.3
*Central Region*			12.0
Zambezia	14.1	15.6	8.7
Tete	9.2	13.9	4.9
Manica	9.2	11.8	13.6
Sofala	8.9	8.8	
*South Region*			
Inhambane	2.0	2.1	6.4
Gaza	5.9	6.7	7.4
Maputo Province	4.6	4.8	6.9
Maputo City	12.2	10.1	5.9
Missing	0.3	0.0	0.0
**Obstetric History**			
*Gravidity*			
1	20.1	24.8	Not available
2–4	49.3	54.1	Not available
5+	30.5	21.1	Not available

a Based on 487 women observed in the Quality of Care Study [31] who gave consent to the follow-up interview.

Tables 3 and 4 present validation results, the estimated survey-based prevalence of indicators, and the inflation factor for indicators with cell sizes sufficient for analysis. Table 3 includes recommended indicators based on one or both of our acceptability criteria. Table 4 includes indicators that cannot be recommended based these same criteria. In Tables 3 and 4 the estimated prevalence of indicators was based on their “true” prevalence as measured in the entire sample of Quality of Care Study observations (i.e., not just those that were matched with follow-up interviews) and the sensitivity and specificity resulting from this study. “Don't know” responses constituted a small percentage of responses in the Quality of Care and follow-up Study and were treated as “No. ” Most indicators were based on a single question, but some were composite indicators based on a combination of responses to two or more questions. Although 525 women consented to observation in the Quality of Care Study [31], differing numbers of women were observed during the different stages of the peripartum period, shown in the varying Ns for the true prevalence from the Quality of Care Study [31] and for AUC, which was based on cases of women observed during that indicator-specific period matched to their follow-up responses. In total, 27 of 34 indicators had sufficient numbers in all cells of the 2×2 table for the validation exercise. Table 5 includes indicators which could not be assessed due to small cell sizes.

**Table 3:** Summary of validation results: Recommended indicators.

Variable	Sensitivity (95% CI) of Follow Up Responses	Specificity (95% CI) of Follow Up Responses	N from Quality of Care Study	True Prevalence (%) among Those Observed in Quality Care Study	Population-Based Survey Estimate (%) Based on Sensitivity, Specificity	N for AUC Analysis	AUC	Inflation Factor (IF)	Recommend? Y/N (Selection Criteria: AUC>0.6 or 0.75<IF<1.25)
Woman was encouraged to have a companion during labor OR delivery	0.49 (0.37–0.61)	0.74 (0.67–0.81)	525	33.6	33.8	212	0.62 (0.55–0.69)	1.00	Y (Both criteria)
Woman had a companion present during the labor OR delivery	0.44 (0.33–0.54)	0.78 (0.71–0.84)	440	31.4	29.1	263	0.61 (0.55–0.66)	0.93	Y (Both criteria)
Woman delivered in a hospital (versus a health center)	0.81 (0.75–0.87)	0.94 (0.90–0.98)	525	54.1	46.6	304	0.88 (0.84–0.91)	0.86	Y(Both criteria)
Newborn was placed skin to skin on mother's chest	0.60 (0.52–0.69)	0.69 (0.62–0.76)	508	42.5	43.5	297	0.65 (0.59–0.70)	1.02	Y (Both criteria)
Woman had her labor augmented with injection	0.67 (0.12–1.00)	0.77 (0.72–0.83)	525	2.4	23.6	264	0.72 (0.51–0.93)	9.82	Y (AUC only)
Woman received fundal pressure before birth of baby	0.63 (0.39–0.87)	0.70 (0.65–0.76)	525	5.1	31.4	285	0.67 (0.55–0.78)	6.16	Y (AUC only)
Woman had her blood pressure taken	0.63 (0.54–0.71)	0.48 (0.38–0.59)	378	59.3	58.2	212	0.57 (0.50–0.63)	0.98	Y (IF only)
Woman was encouraged to ambulate or move around during labor	0.54 (0.46–0.63)	0.63 (0.55–0.72)	455	51.4	45.8	265	0.599 (0.53–0.65)	0.89	Y (IF only)
Woman delivered on her back	0.92 (0.88–0.95)	0 (0–0)	507	96.4	91.9	297	0.46 (0.44–0.47)	0.95	Y (IF only)
Woman received fundal massage after delivery of the placenta	0.56 (0.49–0.62)	0.43 (0.31–0.54)	507	71.0	56.1	289	0.49 (0.43–0.56)	0.79	Y (IF only)
Newborn dried and wrapped in a towel/cloth (among those not placed skin-to-skin with mother – composite indicator)	0.59 (0.48–0.69)	0.43 (0.29–0.56)	312	63.1	58.3	144	0.51 (0.42–0.59)	0.92	Y (IF only)
Newborn immediately dried	0.77 (0.72–0.82)	0.31 (0.12–0.50)	508	89.6	76.2	295	0.54 (0.44–0.63)	0.85	Y (IF only)
Birth with cephalic presentation	0.95 (0.93–0.98)	0 (0–0)	507	97.4	95.5	288	0.56 (0.40–0.72)	0.98	Y (IF only)

**Table 4:** Summary of validation results: Indicators not recommended.

Variable	Sensitivity (95% CI) of Follow Up Responses	Specificity (95% CI) of Follow Up Responses	N from Quality of Care Study	True Prevalence (%) among Those Observed in Quality of Care Study	Population-Based Survey Estimate (%) Based on Sensitivity, Specificity	N for AUC Analysis	AUC	Inflation Factor
Woman asked about her HIV status	0.32 (0.24–0.40)	0.64 (0.52–0.77)	378	75.7	32.9	212	0.48 (0.41–0.55)	0.43
Woman offered HIV test (among women with unknown HIV status)	0.40 (0.00–1.00)	0.58 (0.47–0.70)	378	1.9	13.8	82	0.50 (0.22–0.74)	7.27
Woman draped for privacy during labor	0.60 (0.48–0.72)	0.40 (0.33–0.47)	455	23.3	59.8	261	0.52 (0.45–0.58)	2.56
Woman had more than one health care provider assisting during birth	0.10 (0.02–0.17)	0.87 (0.83–0.91)	507	21.3	12.4	299	0.48 (0.44–0.53)	0.58
Woman received a uterotonic within 3 (a few) minutes after birth of baby	0.38 (0.31–0.45)	0.66 (0.58–0.75)	507	56.8	36.3	289	0.52 (0.46–0.58)	0.64
Woman received controlled cord traction	0.83 (0.77–0.89)	0.25 (0.17–0.32)	507	54.2	79.4	286	0.54 (0.49–0.59)	1.47
Active management of third stage of labor (composite indicator)^a^	0.24 (0.15–0.32)	0.81 (0.76–0.87)	507	31.4	20.2	282	0.52 (0.47–0.58)	0.64
Newborn is placed skin to skin on mother covered with a cloth (composite)	0.56 (0.42–0.69)	0.21 (0.07–0.35)	508	41.7	69.2	92	0.38 (0.29–0.48)	1.66
Newborn is wrapped in a towel/cloth	0.82 (0.74–0.90)	0.14 (0.02–0.26)	508	41.5	84.2	130	0.54 (0.46–0.62)	2.03
Newborn immediately dried, placed skin to skin and covered with a towel/cloth (composite)	0.40 (0.269–0.55)	0.40 (0.25–0.55)	508	38.2	52.5	92	0.40 (0.30–0.50)	1.37
Breastfeeding of newborn initiated within one hour of birth	0.82 (0.72–0.92)	0.25 (0.20–0.31)	508	19.3	75.9	296	0.54 (0.48–0.59)	3.94
Newborn held upside down	0.50 (0.05–0.95)	0.40 (0.34–0.46)	525	2.3	59.7	300	0.49 (0.31–0.68)	25.94
Newborn bathed within one hour (i.e., bathing not delayed)	0.00 (0.00–0.00)	0.99 (0.97–1.00)	507	2.8	1.4	300	0.49 (0.49–0.50)	0.49
Woman for whom birth attendant manually explored uterus after birth of baby	0.62 (0.46–0.79)	0.42 (0.36–0.48)	525	10.1	58.6	288	0.55 (0.46–0.63)	5.80

a Uterotonic (injection) within a few minutes after birth, controlled cord traction AND fundal massage after delivery of placenta.

**Table 5:** Summary of validation results: Indicators that could not be assessed due to small cell size*.

Variable	N from Quality of Care Study	True Prevalence (%) among Those Observed in Quality of Care Study
Woman asked for urine sample upon arrival at the health facility	525	1.6
Woman delivered by cesarean section	525	2.9
Woman with an instrumental birth (forceps, vacuum extraction)	525	2.9
Woman received an episiotomy	507	3.0
Woman slapped, physically mistreated	378	1.0
Woman shouted at or otherwise verbally mistreated	525	1.1
Newborn slapped	525	0.2

Sensitivity and specificity were not analyzed in cases where the n of any cell was <5.

Six indicators had AUC results of 0.60 or greater (Table 3). The most accurately reported responses were to the question on whether the woman delivered in a hospital versus a health center (0.88, 95% CI: 0.84–0.91); her labor was augmented (0.72, 95% CI: 0.51–0.93); fundal pressure was applied before birth of baby (0.67, 95% CI: 0.55–0.78); the newborn was placed skin to skin against the mother (0.65, 95% CI: 0.59–0.70); the woman was encouraged to have a companion during labor or birth (0.62, 95% CI: 0.55–0.69); and the woman had a support person present during labor or delivery (0.61, 95% CI: 0.55–0.66).

The other criterion of acceptable validity was an inflation factor between 0.75 and 1.25. Eleven indicators met this criterion. These were: the woman was encouraged to have a companion during labor/delivery (1.00); newborn was placed skin to skin against the mother (1.02); blood pressure was taken during initial assessment (0.98); the baby was cephalic presentation at birth (0.98); the woman delivered on her back (0.95); the woman had a support person present during labor or delivery (0.93); the newborn was immediately dried and wrapped in a towel (0.92); the woman was encouraged to ambulate or move around during labor (0.89); the woman delivered in a hospital versus health center (0.86); the newborn was immediately dried after birth (0.85); and the woman had fundal massage following delivery of the placenta (0.79).

Four indicators met the criteria for quality reporting based on both AUC and the IF. These were: the newborn was placed skin to skin against the mother, the woman identified her place of birth as a hospital versus a health center, the woman was encouraged to have a companion during labor or birth, and a support person was present during labor or birth.

Of the high-demand, evidence-based indicators tested, blood pressure measurement and two individual components of thermal care (baby was immediately dried and baby was placed skin to skin against the mother) met the criterion for inflation factor.

Two of the three indicators with an inflation factor of >6.0 had a true prevalence of 3% or less (labor augmentation, baby held upside down, fundal pressure applied before birth of baby). Such poor reporting is not surprising for low-prevalence indicators for which even small deviations from 100% in specificity can lead to extreme over-estimation in a survey. Indicators in Table 5 which could not be assessed due to cell size include: women was asked for a urine sample upon arrival at the health facility, cesarean section, instrumental birth, episiotomy, women physically mistreated, women verbally mistreated, and newborn slapped.

## Discussion

This study was able to test the validity of 27 key MNCH coverage indicators. We could not identify another published validation study of mothers' self-report of facility-based interventions delivered around the time of birth that compared women's reports against direct observation of the birth. Given the poor quality of clinical records in low-income settings, use of direct observation of care as the reference standard is a major strength of this study. Additional strengths include: the validity and reliability of clinical observers' observations were confirmed during training, observers were assigned to facilities other than their own places of work, and the home-based follow-up study closely mimicked the conditions of data collection in the DHS and MICS surveys.

Although the eight- to 10-month recall period for the follow-up study is shorter than that for the DHS (up to five years) and MICS (up to two years) surveys, it represents a lengthy recall period not far from the average recall period of a MICS survey and is an improvement over validation studies interviewing women at facility discharge. Furthermore, lengthening the follow-up period would undoubtedly result in greater loss to follow up.

With some caveats, study data are representative of the population of women seeking facility-based care at birth in Mozambique. In the Quality of Care Study [31] all provinces in Mozambique were represented, though the sample somewhat over-represented urban and larger facilities. The response rate of the current study was 63% (lower than the 72% response rate of one published validation study using a lengthy recall period [27]), contributing to wider than anticipated confidence intervals for sensitivity and specificity of the indicators. Respondents to the follow-up study were somewhat more highly educated, younger, and more likely to be urban as compared to nationally representative MICS data. Some of this difference is likely due to the lower than expected response rate (as rural women, women without cell phone access and lower-educated women may be more mobile and difficult to locate). Thus, these results may overstate women's ability to self-report peripartum care if education is positively related to the accuracy of reporting, which was not explored in this paper. Among health facilities with surgical capacity, the sample of births observed missed women directly admitted through the emergency ward and prevented validation of delivery by cesarean section. It is unknown whether the observer's presence influenced women's ability to report on the care they received. Finally, our acceptability criterion of 0.60 for AUC may be considered low, and therefore a study limitation. However, given that so few key survey-based variables relied upon for international and national monitoring have been validated to date and that all of the variables assessed here occurred while women were in labor or shortly thereafter, these authors judged a minimum AUC of 0.60 to be acceptable.

Results from this study vary in comparison with other studies. In a study by Liu et al. in China [29], two indicators similar to those measured in this study included: blood pressure check and providing an HIV test. However, in the Liu study, questioning was about antenatal, not intrapartum, care. Liu et al. found higher sensitivity and lower specificity than the current study for both indicators. The differences may be explained by the fact that the prevalence for each of these screening tests was much higher in the Chinese study, possibly leading women to assume that they had always been performed, or that events during antenatal care were easier to be aware of and to recall than events when in labor.

Pacque et al. [38], whose study included home births, measured two indicators of immediate newborn care that are similar to indicators in the current study: “percent of mothers who breastfeed their infant within one hour of birth” and “percent of mothers whose newborn was immediately warmed (dried) and wrapped after birth.” The sensitivity and specificity of the first indicator was relatively similar to results of the current study—0.88 compared with 0.82 for sensitivity in the current study with very low specificity in both studies. For the second indicator, Pacque et al. found lower sensitivity (0.64 versus 0.81) and higher specificity (0.52 versus 0.14) than the current study. It is unclear why the specificity in the current study is so low. The formulation of the question relating to “newborn immediately dried” in the two studies was slightly different.

Yoder et al. [39], who conducted a qualitative investigation of newborn and postnatal care among mothers in Malawi and Bangladesh detected problems with their understanding of (1) terminology related to newborn thermal care, (2) questions about the timing of events following birth, and (3) questions related to postnatal care health checks. The first two issues may have affected our study results as well as those of Pacque.

One of the indicators of greatest interest tested in this study was “woman received an injection within the first few minutes after birth,” (i.e., received a prophylactic uterotonic against postpartum hemorrhage). Data on this important indicator are sparse and, disappointingly, this indicator cannot be recommended based on our results. It should be noted, however, that this and several other questions in the follow-up study were long, complex, and referred to specific time periods (e.g., before and after delivery of the baby; after delivery of the baby and before delivery of the placenta), all elements of questionnaire design best avoided. Qualitative research may assist in improving the formulation for these questions. We also hypothesize that educating women about key preventive interventions that they should expect to receive at birth may improve reporting by raising awareness of these interventions.

### Recommendations

We recommend that validation studies rely on two methods to assess the validity of selected indicators: sensitivity, specificity, and AUC, and the inflation factor or its mathematical equivalent. Two methods were selected because they are complementary and neither is sufficient alone if the goal is coverage monitoring via population-based surveys. Although high sensitivity and specificity are preferred for all indicators, knowing the estimated survey-based prevalence is also helpful, particularly for indicators of very low prevalence which are likely to be over-estimated without near-perfect specificity. Likewise, in some cases, low sensitivity and specificity cancel out at the population level and may generate acceptable estimates for coverage monitoring purposes, even if not accurate for analysis at the individual level. An example from this study includes the indicator for newborns dried and wrapped in a towel (among those not placed skin-to-skin against the mother's chest), with sensitivity at 0.59, specificity at 0.43, and an inflation factor of 0.92.

We recommend that the 13 indicators which met acceptability criteria should be included in more detailed studies of maternal and newborn care, while noting that researchers should be cautious about measurement of low-prevalence indicators. For example, the prevalence of labor augmentation was only 2.4% in the Quality of Care Study [31]. Although its inflation factor was large, it scored well by the AUC criterion. It is important to monitor this intervention as it is frequently misused in other contexts, particularly in South Asia [40]. In such areas where labor augmentation is more prevalent, this indicator may be more accurately estimated in surveys. Among the high-demand, evidence-based indicators assessed, presence of a support person during labor/delivery and placement of the newborn skin to skin against the mother met both acceptability criteria and are therefore recommended for international survey programs.

The results of this study suggest that there are some aspects of peripartum care that women can report with adequate accuracy. Although Mozambique was selected as representative of high maternal and newborn mortality settings, additional studies with a modified design in other low-income settings are needed to confirm these results. Suggested modifications include: planning for a higher loss to follow up rate (e.g. at least 35%); where possible, improving question formulation for complex questions that relate to very specific time periods; and considering a design in which face-to-face interviews could be divided into two or three arms, with interviews at hospital discharge, at the woman's house after a lengthy interval and at the woman's house at the mid-point between interviews in the first and second arms of the study. Experience from this study suggests that a follow-up period of greater than approximately 12 months may not be feasible, given the effects of an extended period on the loss to follow-up rate. Cell phone penetration and population mobility will ultimately determine what is possible within a given context.

Such a design would allow one to determine if inaccuracy in reporting is due to recall or to the fact that women were never aware that certain procedures were performed. The three-arm design would also allow one to determine whether recall worsens over time. Depending on the results of such studies, data quality on peripartum care may be improved by restricting such detailed survey questions to births in the last two years.

## Supporting Information

Checklist S1 Portuguese version of the questionnaire.
(DOCX)

Checklist S2 English version of the questionnaire.
(DOCX)

## Abbreviations

AUC: area under receiver operating characteristic curve
DHS: Demographic and Health Survey/s
IF: inflation factor
MICS: Multiple Indicator Cluster Survey/s
MNCH: maternal, newborn, and child health
