# Supplementary material for: Measuring Coverage in MNCH: Testing the Validity of Women's Self-Report of Key Maternal and Newborn Health Interventions during the Peripartum Period in Mozambique
Source: PLoS One. 2013 May 7;8(5):e60694. doi: 10.1371/journal.pone.0060694 (PMC3646219; doi:10.1371/journal.pone.0060694)
Supplement: Checklist S1 — Portuguese version of the questionnaire. (DOCX) [file pone.0060694.s001.docx]

**Estudo de indicadores de materno em Moçambique**

| **#** | **Questões** | **Código de Resposta** | **SALTAR PARA** |
| --- | --- | --- | --- |
| 1. **CAPA DO QUESTIONÁRIO** | | | |
|  | CÓDIGO/NOME DO ENTREVISTADADOR | \|___\|___\|___\| |  |
|  | CÓDIGO DA US  *(REGISTAR ANTES DO INÍCIO DA ENTREVISTA)* | \|___\|___\|___\| |  |
|  | NOME DA MULHER  *(PRENCHER ANTES DO INÍCIO DA ENTREVISTA)* |  |  |
|  | CÓDIGO DA MULHER | \|___\|___\|___\| |  |
|  | NOME DO DISTRITO | ………………………………………………………………….. |  |
|  | NOME DA PROVÍNCIA | ………………………………………………………………….. |  |
|  | ÁREA DE RESIDÊNCIA | URBANO………………………………………………..………1  RURAL………………………………………..………………….2 |  |
|  | DATA DA 1ª VISITA AO DOMICÍLIO | \|  \|  \|  \|  \| **1** \| **2** \| \| --- \| --- \| --- \| --- \| --- \| --- \|   D D M M A A |  |
|  | RESULTADO DA 1ª VISITA AO DOMICÍLIO  **PARA COMPLETAR NO FIM DA ENTREVISTA OU NO FIM DA TENTATIVA DE CONTACTO** | ENTREVISTA COMPLETA……………………..…………….1  LOCALIZADA, MAS TEVE QUE REPROGRAMAR….2  RECUSA EM SER ENTREVISTADA………………………..3  NÃO FOI POSSÍVEL LOCALIZAR…………………………..4  ENTREVISTA PARCIALMENTE COMPLETA ……….…5  MULHER FICOU INCAPACITADA…....……………..….6  FALECEU………………………………………….............……7 | FIM  109  FIM  FIM  FIM  FIM  FIM |
|  | DATA DA 2ª VISITA AO DOMICÍLIO | \|  \|  \|  \|  \| **1** \| **2** \| \| --- \| --- \| --- \| --- \| --- \| --- \|   D D M M A A |  |
|  | RESULTADO DA 2ª VISITA AO DOMICÍLIO  **PARA COMPLETAR NO FIM DA ENTREVISTA OU NO FIM DA TENTATIVA DE CONTACTO** | ENTREVISTA COMPLETA……………………..…………….1  ENTREVISTA PARCIALMENTE COMPLETA …………2  RECUSA EM SER ENTREVISTADA …….………………..3  MULHER FICOU INCAPACITADA …..……………….….4 | FIM  FIM  FIM  FIM |
|  | INÍCIO DA ENTREVISTA: USE O RELÓGIO DE 24 HORAS | HORAS……………………………….\|___\|___\|  MINUTOS………………….……...\|___\|___\| |  |
|  | LOCAL DA ENTREVISTA? | EM CASA DA MULHER………………………………………1  OUTRA CASA…………………………………………………….2  OUTRO LUGAR………………………………………………….8  (ESPECIFIQUE) |  |
|  | REGISTE AS COORDENADAS GEOGRÁFICAS DO LOCAL DA ENTREVISTA | LATITUDE .  LONGITUDE . |  |
| Olá. Chamo-me _________________. Sou um entrevistador representando o Ministério de Saúde e o Programa  Integrado de Saúde Materna e Infantil patrocinado por USAID. Estamos realizando um estudo de mulheres que  deram à luz recentemente em Moçambique, para entender melhor o sua experiência de dar a luz. Se você concorda,  eu gostaria de entrevistar-lhe sobre a sua experiencia dando a luz. A entrevista vai durar mais ou menos 30 minutos.  Por favor, saiba que o decisão para participar nesta entrevista é completamente voluntária, e pode desistir em  qualquer altura. Podia sentir algum incómodo durante a entrevista por causa da natureza das perguntas. Podia haver  nenhum benefício directo para você por estar neste estudo unidade sanitária, mais os resultados vão informar os  actividades neste unidade sanitária. Nem o seu nome nem a data dos serviços será fornecido em qualquer dado  partilhado, portanto a sua identidade e qualquer informação sobre você permanecerá completamente confidencial.   Pode telefonar para o investigador principal a Jim Ricca a +258-82-305-3916 caso tenha alguma dúvida ou  preocupação relativamente a este estudo.   Se tiver qualquer pergunta sobre os seus direitos como um participante neste estudo, ou se pensa que não  foi tratada numa forma justa, pode ligar para Dr Francisco Mbofana no Comite Nacional de Bioetica a | | | |

| **2. INFORMAÇÃO GERAL** | | | |
| --- | --- | --- | --- |
|  | Quantos anos tinha na data do seu último aniversário?  (SONDAR CASO A MULHER NÃO SAIBA A SUA IDADE, Caso contrário faça uma estimativa) | IDADE EM ANOS ............................... \|___\|___\| |  |
|  | Alguma vez frequentou uma escola? | SIM……………………….……………………………………….1  NÃO…………………………………….…………………………0 | 204 |
|  | Qual o nível de escolaridade mais elevado que frequentou? | ALFABETIZAÇÃO.....................................................00  PRIMÁRIO EP1........................................................01  PRIMÁRIO EP2........................................................02  SECUNDÁRIO ESG1.................................................03  SECUNDÁRIO ESG2.................................................04  TÉCNICO ELEMENTAR............................................05  TÉCNICO BÁSICO.....................................................06  TÉCNICOMÉDIO......................................................07  CURSOS DE FORMAÇÃODE PROFESSORES…………..08  SUPERIOR................................................................09 |  |
|  | A senhora consegue ler facilmente um jornal, uma carta, ou têm alguma dificuldade ou não sabe completamente ler? | FACILMENTE…………………………..………..……………………..1  COM DIFICULDADES………………………….……….....……….2  NÃO SABE LÊR…………………………………..............……….3 |  |
|  | Neste momento a senhora está solteira, casada, vive maritalmente, é divorciada ou está viúva? | SOLTEIRA.................................................................01  CASADA...................................................................02  VIVE MARITALMENTE..............................................03  DIVORCIADA ...........................................................04  VIÚVA.......................................................................05 | 208  208  208 |
|  | Alguma vez o seu marido frequentou a uma escola? | SIM……………….…………………………….…………………..1  NÃO……………………………………………..………………….2  NÃO SABE........…………………………….………………….0 | 208  208 |
|  | Qual o nível de escolaridade mais elevado que o seu marido frequentou? | ALFABETIZAÇÃO.....................................................00  PRIMÁRIO EP1........................................................01  PRIMÁRIO EP2........................................................02  SECUNDÁRIO ESG1.................................................03  SECUNDÁRIO ESG2.................................................04  TÉCNICO ELEMENTAR............................................05  TÉCNICO BÁSICO.....................................................06  TÉCNICOMÉDIO......................................................07  CURSOS DE FORMAÇÃODE PROFESSORES…………..08  SUPERIOR................................................................09 |  |
|  | Qual é a sua principal ocupação?  (*O tipo de trabalho que frequentemente realiza?)* | DOMÉSTICA..............................................................00  ESTUDANTE..............................................................01  CAMPONESA............................................................02  FUNCIONÁRIA PÚBLICA...........................................03  CONTA PRÓPRIA......................................................04  FUNCIONÁRIA SECTOR PRIVADO.............................05  OUTRA___________________________________08  (ESPECIFIQUE) | 211 |
|  | O seu local de trabalho é fora ou dentro de casa? | DENTRO…………………………………………………………….1  FORA…………………………………………………………..…….2 |  |
|  | A senhora trabalha para alguém da sua família, ou é independente? | TRABALHA PARA MEMBRO DA FAMÍLIA………………..1  TRABALHA PARA OUTRA PESSOAS………………..….……2  INDEPENDENTE…………………………………..………….…….3 |  |
|  | Tem em sua casa:  Fonte de água (onde tira água)  Eletricidade?  Telefone celular? | SIM NÃO    1 2    1 2  1 2 |  |
|  | Vive em casa própria ou casa arrendada? | CASA PRÓRIA……………………………………......…….…….1  CASA ARRENDADA………………………………………….…..2  OUTRO (ESPECIFIQUE)______________________3 |  |

| **3. REPRODUÇÃO** | | | | |
| --- | --- | --- | --- | --- |
|  | Ficou grávida depois do parto que teve em Setembro/Outubro de 2011? | SIM………………………………………………..……………….1  NÃO……………………………………….……………………….0 | | 303 |
|  | Qual foi o resultado desta gravidez mais recente (desde Setembro / Outubro 2011)? | AINDA ESTÁ GRÁVIDA.………..……………...……..1  ABORTO ESPONTÂNEO………..……………...……..2  ABORTO PROVOCADO.………………………………..3  NATIMORTO………………………………………….…...4  NASCIMENTO VIVO….………………………………….5 | |  |
|  | Quantos filhos que deu à luz vivem consigo? | NÚMERO DE FILHOS VIVENDO COM A MULHER  \|____\|____\| | |  |
|  | Quantos filhos deu à luz não vivem consigo em casa? | NÚMERO DE FILHOS QUE NÃO VIVEM COM A MULHER  \|____\|____\| | |  |
|  | Quantos filhos que deu à luz nasceram vivos, mas falaceram depois? | Número de filhos nascidos vivos, mas depois morreram  \|____\|____\| | |  |
|  | Quanto nados-mortos já teve, isto é bebés que nasceram mortos | NÚMERO DE NATIMORTOS  \|____\|____\| | |  |
|  | Quantas vezes já esteve grávida, mas perdeu a gravidez antes do parto? | Número de gravidezES PERDIDAS antes Do parto \|____\|____\| | |  |
|  | Assim, o número total de vezes que já esteve grávida é:  *(INSERIR O SOMATÓRIO DOS VALORES DAS QUESTÕES PERGUNTAS 303-307).* | TOTAL DE GRÁVIDEZES \|____\| | |  |
|  | 308A. Está correto? Caso não esteja correto revise as questões de 303-307, CORRIJA O ERRO E REGISTE CORRECTAMENTE EM 308, E DEPOIS CIRCULE, 1-SIM. Caso não esteja correta DEVIDO A EXIXTÊNCIA DE GÊMEOS / nascimento múltiplo, MANTENHA A RESPOSTA E CIRCULE, 1-SIM CORRECTO | SIM, CORRECTO……………………………………………….1 | |  |
|  | Agora eu tenho algumas perguntas sobre o seu parto em setembro / outubro de 2011. ***Enfatize para as entrevistadas que tiveram parto em Setembro / Outubro 2011 que estas perguntas são sobre a gravidez que terminou em setembro / outubro de 2011 E NÃO A SUA GRAVIDEZ MAIS RECENTE .*** | | |  |
|  | O seu parto em setembro / outubro de 2011, foi de um único nascimento ou um nascimento múltiplo (gêmeos)? | | ÚNICO BEBÉ…………………………………….…………….1  MULTIPLO………………………………………….………….2 |  |
|  |  |  |  |  |
|  | Como termou o parto de Setembro / Outubro de 2011? O seu bebé(s) nasceu vivo ou morto? **Se a mulher TEVE GÊMEOS, REGISTE AS respoSTAS apenas DO 2º GÉMEO** | | NASCIDO VIVO………………………………………………..1 NADO MORTO……………………………….……..………..2 | 315 |
|  | Sabe se o seu bebé morreu antes do início das dores de trabalho de parto de Setembro / Outubro de 2011? | | SIM…………………………………………….……………………1  NÃO…………………………………………………………………2  NÃO SABE.......................................…………………8 |  |
|  | Depois do parto, foi-lhe mostrado o seu bebê que nasceu em Setembro / Outubro 2011? | | SIM…………………………………………….……………………1  NÃO…………………………………………………………………2  NÃO SABE.......................................…………………8 |  |
|  | A pele do seu bebé estava intacta ou descamada | | PÊLE INTACTA………………………..……………………….1  PÊLE DESCAMADA………………….………………………2  NÃO SABE……..………………………..……………………..8 |  |
|  | 1. O bebé foi dado um nome? | | SIM…………………………………………….……………………1  NÃO…………………………………………………………………2 | (Se 311=Nado morto **E** 315a=Nao, SALTAR para 317) |
|  | 1. Qual foi o nome atribuído ao seu bebê? | | NOME DO BEBÊ_________________________1  NÃO DERAM NOME AO BEBÊ……………………….…8 |  |
|  | O **[NAME DO BEBÊ]** ainda está vivo? | | SIM…………………………………………….……………………1  NÃO…………………………………………………………………0 | 318 |
|  | Quantos anos **[NOME DO BEBÊ]** tinha (ele/ela quando faleceu)?  RESPONDA EM DIAS SE FALECEU ANTES DE 1 MÊS; RESPONDA EM MESES SE FALECEU DEPOIS DE 1 ANO+ MESES | | DIAS ...........................\|__\|__\|    MESES....................... \|__\| __\| |  |
|  | É **[NOME DO BEBÊ]** rapaz ou rapariga? | | RAPAZ………………………………………………………………..1  RAPARIGA……………………………………………………..….2 |  |
|  | Tem um cartão de saúde com suas informações (da mulher)? | | SIM……………………………………………………..…………..1  NÃO………………………………………………………………...0 | 321 |
|  | Posso ver o seu cartão de saúde? RESPONDER SIM SE a mulher mostra-lhe o CARTÃO | | SIM……………………………………………………..…………..1  NÃO…………………………………………………………………0 |  |
|  | Tem cartão de saúde do/a [**NOME]**?  *(Nom: Refere-se ao nome da criança)* | | SIM……………………………………………………..…………..1  NÃO…………………………………………………………………0 | 401 |
|  | Posso ver o cartão da (o) **[NOME]** | | SIM……………………………………………………..…………..1  NÃO…………………………………………………………………0 |  |

| **4. CUIDADOS DE SAÚDE DURANTE O PARTO EM SETEMBRO/OUTUBRO DE 2011** | | | | |
| --- | --- | --- | --- | --- |
| Agora tenho algumas questões sobre os cuidados de saúde que recebeu. Vou começar por questões sobre suas gravidezes anteriores e depois vou perguntar-lhe sobre cuidados recebidos durante a sua gravidez e parto ocorrido em Setembro/Outubro de 2011. Nós entendemos que algumas das questões poderão ser difíceis para uma mulher responder. Contudo, se não souber a resposta ou não se lembrar, simplesmente informe-me. | | | | |
|  | Alguma vez teve parto por cesariana? Ou seja, um parto onde eles cortaram sua barriga para tirar o bebé? | SIM…………………………………………….……………………1  NÃO…………………………………………………………………0  NÃO SABE/NÃO SE LEMBRA...........…………………8 | |  |
|  | Teve alguma consulta pré-natal para a gravidez que terminou em Setembro/Outubro de 2011? | SIM…………………………………………….……………………1  NÃO…………………………………………………………………0 | | 406 |
|  | Quando foi a consulta pré-natal para a gravidez que terminou em Setembro/Outubro de 2011. Quem foi que a atendeu ou assistiu-lhe nessa consulta  LISTAR TODOS OS CLÍNICOS | SIM NÃO  MÉDICO ……………….....................1 2  ENFERMEIRA ……………..…..........…1 2  PARTEIRA TRADICIONAL………......1 2  OUTRO VOLUNTÁRIO DA  COMUNIDADE………………..….........1 2 | |  |
|  | Onde recebeu cuidados pré-natais para a sua gravidez que terminou em Setembro /Outubro de 2011?  LISTAR TODAS AS QUESTÕES APLICÁVEIS  (*Múltipla resposta*) | PÚBLICO: SIM NÃO  HOSPITAL …………………………............1 2  CENTRO DE SAÚDE….……………........ 1 2  POSTO DE SAÚDE…………………......….1 2  CLÍNICA ...............…………............…...1 2  OUTRO_______________________________  (ESPECIFICAR)  PRIVADO:  HOSPITAL PRIVADO ………...............1 2  CENTRO DE SAÚDE PRIVADO……….1 2  OUTRO SECTOR PRIVADO……………..1 2  ____________________________________  (ESPECIFICAR)  NÃO GOVERNAMENTAL (ONG):  UNIDADE SANITÁRIA (ONG)…………1 2 | |  |
|  | Quantas vezes recebeu cuidados pré-natais para a gravidez que terminou em Setembro / Outubro 2011? | NÚMERO DE VISITAS PRÉ-NATAIS \|__\| __\|  NÃO SABE/NÃO SE LEMBRA……………..........08 | |  |
|  | Where did you go to deliver the pregnancy that ended in Set/Out 2011?  Se não for capaz de identificar se foi público, privado ou ONG, anote o nome da US: | PÚBLICO: SIM NÃO  HOSPITAL ………………………….............1 2  CENTRO DE SAÚDE….……….……........ 1 2  POSTO DE SAÚDE…………….……......….1 2  CLÍNICA ...............………….............…...1 2  OUTRO_______________________________  (ESPECIFICAR)  PRIVADO:  HOSPITAL PRIVADO ………...............1 2  CENTRO DE SAÚDE PRIVADO……….1 2  OUTRO SECTOR PRIVADO……………..1 2  ____________________________________  (ESPECIFICAR)  NÃO GOVERNAMENTAL (ONG):  UNIDADE SANITÁRIA (ONG)……….…1 2 | |  |
|  | Depois que chegou à unidade sanitária para o nascimento de [NOME DO BEBÊ] em setembro / outubro 2011, alguém mediu a sua pressão arterial? | SIM…………………………………………….……………………1  NÃO…………………………………………………………………2  NÃO SABE/NÃO SE LEMBRA...........…………………8 | |  |
|  | Alguém pediu amostra de sua urina para análises | SIM…………………………………………….……………………1  NÃO…………………………………………………………………0  NÃO SABE/NÃO SE LEMBRA...........…………………8 | |  |
|  | *Agora tenho algumas questões sobre testagem de VIH (HIV). Contudo, saiba que não iremos questionar-lhe sobre o seu estado serológico.*  Enquanto estava na unidade sanitária para o nascimento do [NOME(S)] em Setembro/Outubro, alguém perguntou-lhe qual era o seu estado serológico? | SIM…………………………………………….……………………1  NÃO…………………………………………………………………0  NÃO SABE/NÃO SE LEMBRA...........…………………8 | |  |
|  | Enquanto estava na unidade sanitária para o nascimento do [NOME(S)] em Setembro/Outubro, alguém ofereceu-lhe a possibilidade de fazer o teste de VIH (HIV)? | SIM…………………………………………….……………………1  NÃO…………………………………………………………………0  NÃO SABE/NÃO SE LEMBRA...........…………………8 | |  |
|  | Enquanto estava na unidade sanitária para o nascimento do [NOME(S)] em Setembro/Outubro, alguém faz-lhe o teste de VIH (HIV)? | SIM…………………………………………….……………………1  NÃO…………………………………………………………………0  NÃO SABE/NÃO SE LEMBRA...........…………………8 | |  |
|  | Para o nascimento do [NOME(S)] em Setembro/Outubro de 2011, teve seu início de trabalho de em casa, à caminho para a unidade sanitaria ou nunca entro em trabalho de parto? | EM CASA…………….……………………………….…..…….1  A CAMINHO PARA A US…….……………………………2  UMA VEZ CHEGADO A US……………………………….3  NUNCA ENTRO EM TRABALHO DE PARTO……...4  NÃO SABE/NÃO SE LEMBRA ……......….………..….8 | |  |
|  | Para o nascimento de [NOME / (S)] em Setembro / Outubro de 2011, o seu trabalho de parto começou espontaneamente ou alguém fez alguma coisa para começar o seu trabalho? | TRABALHO DE PARTO ESPONTÂNEO………..…….1  alguém fez alguma coisa para  começar o trabalho DE PARTO…………………2  NÃO SABE/NÃO SE LEMBRA .......…………………… 8 | | 415  415 |
|  | Para o nascimento de [NOME / (S)] em Setembro / Outubro de 2011, o que foi feito para o trabalho de parto? | CATATERIZAÇÃO DA VEIA PARA ADMINISTRACAO DO SORO…………………..…………………………..1  OUTRO)……………………………………….……………..……..8  _____________________________________  (ESPECIFICAR | |  |
|  | Para o nascimento de [NOME / (S)] em Setembro / Outubro de 2011, alguém ofereceu-lhe a oportunidade de ter uma companhia durante o trabalho e parto? | SIM…………………………………………….……………………1  NÃO…………………………………………………………………0  NÃO SABE/NÃO SE LEMBRA.………………………..…8 | | 418  418 |
|  | Para o parto em Setembro / Outubro de 2011, escolheu em ter alguém para lhe fazer companhia? | SIM…………………………………………….……………………1  NÃO…………………………………………………………………0  NÃO SABE/NÃO SE LEMBRA.………………..…………8 | | 418  418 |
|  | Para o parto em Setembro / Outubro de 2011, a quem escolheu como sua companhia? | MÃE………………………………………………..……….……..1  SOGRA……………………………..………….............……..2  IRMÃ……………………………………………….………….…..3  CUNHADA..........................................................4  PARTEIRA TRADICIONAL…………….……….………..…5  OUTRO MEMBRO DE FAMÍLIA………….……….…….6  OUTRO (ESPECIFICAR)………………..……….…………..8  417a)______________________________________ | |  |
|  | Para o parto em Setembro / Outubro de 2011, alguém perguntou-lhe se queria se levantar e caminhar ao redor enquanto estava em trabalho de parto? | SIM…………………………………………….……………………1  NÃO…………………………………………………………………0  NÃO SABE/NÃO SE LEMBRA...........…………………8 | |  |
|  | Para o parto em Setembro / Outubro de 2011, teve iniciativa, ou preferiu em levanter-se e dar voltas enquanto estava em trabalho de parto? | SIM…………………………………………….……………………1  NÃO…………………………………………………………………0  NÃO SABE/NÃO SE LEMBRA...........…………………8 | |  |
|  | Para o parto em Setembro / Outubro de 2011, alguém perguntou-lhe se queria beber alguns líquidos ou comer algo enquanto estava em trabalho de parto? | SIM…………………………………………….……………………1  NÃO…………………………………………………………………0  NÃO SABE/NÃO SE LEMBRA...........…………………8 | |  |
|  | Para o parto em Setembro / Outubro de 2011, teve iniciativa, ou preferiu beber alguns líquidos ou comer algo enquanto estava em trabalho de parto? | SIM…………………………………………….……………………1  NÃO…………………………………………………………………0  NÃO SABE/NÃO SE LEMBRA...........…………………8 | |  |
|  | Para o parto em Setembro / Outubro de 2011, teve privacidade enquanto estava em trabalho de parto, isto é tinha cortinas em sua volta? | SIM…………………………………………….……………………1  NÃO…………………………………………………………………0  NÃO SABE/NÃO SE LEMBRA...........…………………8 | |  |
|  | Para o parto em Setembro / Outubro de 2011, algo foi feito para acelerar ou reforçar o trabalho de parto? | SIM…………………………………………….……………………1  NÃO…………………………………………………………………0  NÃO SABE/NÃO SE LEMBRA...........…………………8 | | 425  425 |
|  | Para o parto em Setembro / Outubro de 2011, o que foi feito para acelerar ou reforçar o trabalho de parto? | RECEBEU UMA INJECÇÃO DURANTE O  TRABALHO DE PARTO…………………………………...…1  MEDICAMENTO DE LINHA IV ADMINISTRADO DURANTE O TRABALHO DE PARTO.....................2  OUTRO (ESPECIFICAR)…………………………..…….……8  ________________________________________ | | 426 |
|  | Para o parto em Setembro / Outubro de 2011, recebeu alguma injecção durante o trabalho de parto, isto é antes do nascimento do seu bebê? | SIM…………………………………………….……………………1  NÃO…………………………………………………………………0  NÃO SABE/NÃO SE LEMBRA...........…………………8 | |  |
|  | Para o parto em Setembro / Outubro de 2011, foi coberta por uma cortina, ou estava descoberta ou exposta à pessoas a sua volta? | SIM, COBERTA COM CORTINAS………………….……….1  NÃO COBERTA..………………….…………………..………….0  NÃO SABE/NÃO SE LEMBRA ………..……………..………8 | |  |
|  | Para o parto em Setembro / Outubro de 2011, como estava posicionado o bebê no seu útero antes do nascimento? Estava com a cabeça virada para baixo, pés para baixo, ou estava atravessado no útero.  (SE FORAM GÊMEOS, ME REFIRO AO SEGUNDO) | CABEÇA VIRADA PARA BAIXO………………….........1  PÉS PARA BAIXO…………………………………….......….2  TRANSVERSAL/ATRAVESSADO NO ÚTERO……….3  NÃO SABE/NÃO SE LEMBRA ………………......….…..8 | |  |
|  | Para o parto em Setembro / Outubro de 2011, o professional de saúde perguntou-lhe qual era a posição que gostaria de estar durante o trabalho de parto ou para o parto do seu bebê? | SIM…………………………………………….……………………1  NÃO…………………………………………………………………0  NÃO SABE/NÃO SE LEMBRA...........…………………8 | |  |
|  | Para o parto em Setembro / Outubro de 2011, quêm foi que a assistiu?  LISTA TODOS OS PRESENTES | MÉDICO …….…………..............A  ENFERMEIRA...……………......…C  PARTEIRA TRADICIONAL........D  OUTRO…………………………….....E  NINGUÉM……….…………………..F  (ESPECIFICAR)  _______________________________________ | |  |
|  | O [NOME do bebê] foi parto por cesariana? Ou seja, um parto onde eles cortaram sua barriga para tirar o bebê? | SIM…………………………………………….……………………1  NÃO…………………………………………………………………0  NÃO SABE/NÃO SE LEMBRA...........…………………8 | | 433  433 |
|  | Se sim, teve a sua cesariana antes do trabalho de parto ou depois que o trabalho de parto iniciou? | ANTES DE INÍCIO DO TRABALHO DE PARTO……….1  DEPOIS DE INÍCIO DO TRABALHO DE PARTO ….….2  NÃO SABE/NÃO SE LEMBRA..............…………………0 | |  |
|  | Qual foi a razão do seu parto ter sido por cesariana? | O médico/enfermeira disse me que devia ser...01  Eu estava sangrando……………………………….….…...02  O bebê estava preso …………...............…...........…03  Eu estava com dores de trabalho de parto  por muito tempo ……………............................…...04  O bebê estava na posição incorrecta………….…....05  Eu estava doente………………………………………….....06  Meu útero rompeu…………………………………….......07  Houve problemas com o bebê ………..........……...08  Não havia nenhuma razão médica……………...…..10  Não Sei….……………………….......………………….…..….00 | | PASSAR PARA 435 |
|  | O parto foi com ajuda de um forcep (isto é, um instrumento para ajudar a puxar o bebê para fora) ou aspiração para ajudar a puxar o bebê para fora)  (SE FOREM GÊMEOS REFERIR PARA O 2^ND^) | SIM FORCEPS ……………………………………………...…1  SIM VENTOSA….………………………………………....…2  NÃO…………………………………………………………..……3  NÃO SABE/NÃO SE LEMBRA ……..………….……..…8  NA (Parto por cesariana) ………………………….88 | |  |
|  | Para o parto em Setembro / Outubro de 2011, Em que posição estava quando deu o parto de [NOME(S)]? Isto é, estava de costas, com suas mãos apoiadas aos joelhos, de cócoras ou em outra posição? | DE COSTAS………………………………………..………..1  MÃOS APOIADAS AOS JOELHOS…… ……………2  CÓCORAS………….……………………………..…….….3  OUTRA………….…………………….……………..………8  _______________________________________  ESPECIFICAR | |  |
|  | Para o parto de Setembro / Outubro de 2011, pouco antes de ter o seu bebê, alguém cortou a abertura de sua vagina (episiotomia) para dar mais espaço para a cabeça do bebê? | SIM…………………………………………….……………………1  NÃO…………………………………………………………………0  NÃO SABE/NÃO SE LEMBRA...........…………………8 | |  |
|  | **VER A CONSISTÊNCIA COM 430. SE O PARTO TER SIDO POR CESARIANA, SALTAR DIRECTAMENTE PARA A PERGUNTA 449.** | | |  |
|  | Para o parto de Setembro / Outubro de 2011 quando chegou a altura prestes a ter o parto, alguém ficou por cima e aplicou pressão sobre o seu útero? | SIM…………………………………………….……………………1  NÃO…………………………………………………………………0  NÃO SABE/NÃO SE LEMBRA...........…………………8 | |  |
|  | Para o parto de Setembro / Outubro de 2011, quando [NOME/S] nasceu, Qual foi a parte do corpo que saiu primeiro? Cabeça, pés, ou uma outra parte?  (SE FOREM GÊMEOS REFERIR PARA O 2^ND^) | CABEÇA PRIMEIRO.……………………………………1  PÉS PRIMEIRO……………………………………………2  BRAÇO PRIMEIRO ……………………..…..…..….…3  NÃO SABE/NÃO SE LEMBRA….…………………..8 | |  |
|  | Agora tenho algumas questões sobre o seu bebê, logo após o nascimento dele(a) em Setembro / Outubro de 2011  **(SE FOREM GÊMEOS RESPONDER PARA O 2^ND^)** | | |  |
|  | Para o parto de Setembro / Outubro de 2011. Agora, tenho algumas questões referentes ao período *logo após ao parto de NOME(S )*. Nos primeiros minutos após o parto de seu bebê, alguém deu-lhe uma injecção na coxa ou na nádega? | | SIM…………………………………………….……………………1  NÃO…………………………………………………………………0  NÃO SABE/NÃO SE LEMBRA...........…………………8 |  |
|  | Para o parto de Setembro / Outubro de 2011. *Logo após ao parto de NOME(S )*. Nos primeiros minutos depois do parto de seu bebê, alguém deu-lhe medicação por via intravenosa (através de um tubo colocado no seu braço)? | | SIM…………………………………………….……………………1  NÃO…………………………………………………………………0  NÃO SABE/NÃO SE LEMBRA...........…………………8 |  |
|  | Para o parto de Setembro / Outubro de 2011. *Logo após ao parto de NOME(S )*. Nos primeiros minutos depois do parto de seu bebê, alguém deu-lhe comprimidos para tomar/engolir ou manter na sua boca? | | SIM…………………………………………….……………………1  NÃO…………………………………………………………………0  NÃO SABE/NÃO SE LEMBRA...........…………………8 |  |
|  | Para o parto de Setembro / Outubro de 2011. *Logo após ao parto de NOME(S )*. Nos primeiros minutos logo após o parto de seu bebê, alguém pós comprimidos no seu ânus? | | SIM…………………………………………….……………………1  NÃO…………………………………………………………………0  NÃO SABE/NÃO SE LEMBRA...........…………………8 |  |
|  | Consegue se lembra se recebeu esta [injecção / medicação] antes da saída da placenta? | | SIM ……………………………………………………..……………1  NÃO………………………………………………………..……..…0  PLACENTA SAÍU LOGO DEPOIS DO BEBÊ……........3  NÃO SABE/NÃO SE LEMBRA ..........………………..…8 |  |
|  | Para o parto de Setembro / Outubro de 2011, depois do nascimento de [NOME(S)], o profissional de saúde ajudou-a tirar a placenta, ou seja, ela/ele pós a mão com firmeza em seu abdómen inferior com uma mão e segurou o cordão umbilical na outra mão? | | SIM ……………………………………………………..……………1  NÃO………………………………………………………..……..…0  PLACENTA SAÍU IMEDIATAMENTE  DEPOIS DO NASCIMENTO SEM  ASSISTÊNCIA …………………..........................…..….3  NÃO SABE/NÃO SE LEMBRA ..........…………………8  NA, parto por cesariana...................................99 |  |
|  | Para o parto de Setembro / Outubro de 2011. *Logo após ao parto de NOME(S ). Nos primeiros minutos depois da saída da placenta*, alguém deu-lhe injecção na coxa? | | SIM…………………………………………….……………………1  NÃO…………………………………………………………………0  NÃO SABE/NÃO SE LEMBRA...........…………………8 |  |
|  | Para o parto de Setembro / Outubro de 2011. *Logo após ao parto de NOME(S ). Nos primeiros minutos depois da saída da placenta*, alguém deu-lhe compridos para tomar ou manter na sua boca? | | SIM…………………………………………….……………………1  NÃO…………………………………………………………………0  NÃO SABE/NÃO SE LEMBRA...........…………………8 |  |
|  | Para o parto de Setembro / Outubro de 2011. *Logo após ao parto de seu bebê*, o profissional de saúde fez massagem em baixo do seu abdómen para ajudar a contrair o útero? | | SIM…………………………………………….……………………1  NÃO…………………………………………………………………0  NÃO SABE/NÃO SE LEMBRA...........…………………8 |  |
| 446A. Para o parto de Setembro / Outubro de 2011. *Logo após ao parto de NOME(S ). Nos primeiros minutos depois da saída da placenta*, o profissional de saúde fez massagem em baixo do seu abdómen para ajudar a contrair o útero? | | | SIM…………………………………………….……………………1  NÃO…………………………………………………………………0  NÃO SABE/NÃO SE LEMBRA...........…………………8 |  |
|  | Quando o [NOME(S)] nasceu em Setembro/Outubro de 2011, Qual foi o instrumento usado para cortar o cordão umbilical? | | LÂMINA NOVA………………..….………...............…1  LÂMINA USADA……………………………….…….…....2  TESOURA………………………………….………….……….3  FIO/LINHA………………………….......……..…………...4  OUTRO (ESPECIFICAR)……………………………….….5  ________________________________________  NÃO SABE/NÃO SE LEMBRA ……………..………….8 |  |
|  | Para o parto de Setembro / Outubro de 2011, o profissional de saúde que assistiu o parto pôs a mão dele dentro do seu útero depois do parto do seu bebê? | | SIM…………………………………………….……………………1  NÃO…………………………………………………………………0  NÃO SABE/NÃO SE LEMBRA...........…………………8 |  |
|  | O seu bebé foi seco com uma toalha imediatamente após a sua/seu nascimento, dentro de alguns minutos do parto?  (SE FOREM GÊMEOS REFERE-SE AO 2^ND^) | | SIM…………………………………………….……………………1  NÃO…………………………………………………………………0  NÃO SABE/NÃO SE LEMBRA...........…………………8 |  |
|  | Alguém pôs o seu bebê no seu peito, em contacto com a sua pele, imediatamente depois do parto?  (SE FOREM GÊMEOS REFERE-SE AO 2^ND^) | | SIM…………………………………………….……………………1  NÃO…………………………………………………………………0  NÃO SABE/NÃO SE LEMBRA...........…………………8 | 453  453 |
|  | O seu bebé estava enrolado por uma pano enquanto estava deitado no seu peito em contacto com sua pele? | | ENROLADO EM UM PANO………………………………1  BEBÊ NU EM CONTACTO COM A PÊLE..……….…2  NÃO SABE/NÃO SE LEMBRA .......……………………0 | 454  454 |
|  | (Se nu em contacto com sua pele), foi o seu bebê coberto com uma toalha ou pano enquanto estava deitado? | | SIM…………………………………………….……………………1  NÃO…………………………………………………………………0  NÃO SABE/NÃO SE LEMBRA...........…………………8 | 454  454  454 |
|  | Foi seu bebê envolvido em uma toalha ou um pano imediatamente após o nascimento? | | SIM…………………………………………….……………………1  NÃO…………………………………………………………………0  NÃO SABE/NÃO SE LEMBRA...........…………………8 |  |
|  | Depois que seu bebê nasceu, algum de seus atendentes segurou o bebê de cabeça para baixo?  (SE FOREM GÊMEOS REFERE-SE AO 2^ND^) | | SIM…………………………………………….……………………1  NÃO…………………………………………………………………0  NÃO SABE/NÃO SE LEMBRA...........…………………8 |  |
|  | Depois que seu bebê nasceu, algum de seus atendentes bateu suavemente seu bebê para que ele chorasse?  (SE FOREM GÊMEOS REFERE-SE AO 2^ND^) | | SIM…………………………………………….……………………1  NÃO…………………………………………………………………0  NÃO SABE/NÃO SE LEMBRA...........…………………8 |  |
|  | Alguêm pesou o [NOME] logo após o nascimento?  (SE FOREM GÊMEOS REFERE-SE AO 2^ND^) | | SIM…………………………………………….……………………1  NÃO…………………………………………………………………0  NÃO SABE/NÃO SE LEMBRA...........…………………8 | 459  459 |
|  | Qual foi o peso do[NOME]?  (SE FOREM GÊMEOS REFERE-SE AO 2^ND^) | | PESO EM GRAMAS: \|___\|___\|____\|____\| |  |
|  | Indicar onde obteve o peso do bebé | | ATRAVÉS DO CARTÃO DE SAÚDE…………………1  RESPOSTA DADA PELA MÃE…………………..……2 |  |
|  | Para o parto de Setembro / Outubro de 2011, O/A [NAME] nasceu no período normal de gestação, prematuro ou tardio? | | TERMO (Á TEMPO)………………………………………1  PRE-TERMO (EARLY)..…………………………………..2  TARDE ………………………………………………………….3  NÃO SABE/NÃO SE LEMBRA …………….……..…..0 |  |
|  | Para o parto de Setembro / Outubro de 2011, se lembra há quantas semanas estava grávida quando deu o parto da/o [NOME]? | | SEMANAS \|___\|___\|  NÃO SABE, NÃO SE LEMBRA....……..00 |  |
|  | **VERIFICAR AS QUESTÕES 310 E 311. SE FÔR UMA NADO MORTO, SALTAR PARA 463** | | |  |
|  | Amamentou O/A [NOME]  (SE FOREM GÊMEOS REFERE-SE AO 2^ND^) | | SIM…………………………………………….……………………1  NÃO…………………………………………………………………0 | 463 |
|  | Amamentou o/a O/A [NAME]  Dentro da primeira hora depois do nascimento?  (SE FOREM GÊMEOS REFERE-SE AO 2^ND^) | | SIM…………………………………………….……………………1  NÃO…………………………………………………………………0  NÃO SABE/NÃO SE LEMBRA...........…………………8 |  |
|  | A Senhora ou alguém deu alguma coisa para comer ou beber ao bebê dentro da primeira hora depois do parto?  (SE FOREM GÊMEOS REFERE-SE AO 2^ND^) | | SIM…………………………………………….……………………1  NÃO…………………………………………………………………0  NÃO SABE/NÃO SE LEMBRA...........…………………8 |  |
|  | Depois de quanto depois do nascimento o seu bebê teve o primeiro banho?  (SE FOREM GÊMEOS REFERE-SE O 2^ND^) | | DENTRO DE 1 HORA……………………………..……….…1  1-5 HORAS …………………….....………………..………….2  6-24 HORAS ……………………………....……..….………..3  2-3 DIAS………………………………….......……….………..4  DEPOIS DE 3 DIAS……………………………………..……..5  NÃO SABE/NÃO SE LEMBRA …………...................8 |  |
|  | Quantas noites permaneceu (dormiu) na unidade sanitária depois do parto (depois de ter o seu bebé)? | | NOITES PERMANECIDAS NA US …………………\|___\|  (Marcar “0” se não tiver permanecido nenhuma noite)  NÃO SABE/NÃO SE LEMBRA…………………………999 |  |
|  | A Senhora sabe a data de nascimento de/a [NOME DO BEBÉ]? | | SIM…………………………………………….……………………1  NÃO…………………………………………………………………0 |  |
|  | Qual foi a data de nascimento de [NOME]? SE FOREM GÊMEOS, REFERE-SE AO 2^ND^ GÊMEO | | DATA DE NASCIMENTO DO BEBÊ   \|  \|  \|  \|  \|  \|  \| \| --- \| --- \| --- \| --- \| --- \| --- \|   D D M M A A |  |
|  | Durante a sua estadia na unidade de saúde, alguém tratou-lhe mal fisicamente? Ou seja, se alguém bateu em, golpeou-lhe, ameaçou-a fisicamente ou de qualquer outra forma que causou-lhe dano físico?  *(POR FAVOR SELECIONE TODAS ALTERNATIVAS APLICÁVEIS)* | | SIM, AGREDIU-ME………………...................…………1  SIM, BOFETADA……………………………………...……….2  SIM, OUTRO (ESPECIFICAR)  ______________________________________3 NÃO TRATOU-ME MAL FISICAMENTE…………..…4  NÃO SABE/NÃO SE LEMBRA ........…………………..0 |  |
|  | Durante a sua estadia na unidade de saúde, alguém tratou-lhe mal verbalmente? Ou seja, se alguém ameaçou-a verbalmente ou gritou com ?  *(POR FAVOR SELECIONE TODAS ALTERNATIVAS APLICÁVEIS)* | | SIM, AMEAÇOU…………………………………….…….….1  SIM, GRITOU…………………………………………………..2  SIM, OUTRA (ESPECIFIQUE)…………………………...3  ______________________________________ NÃO MAUS TRATOS VERBAL……………….……..…..4  NÃO SABE/NÃO SE LEMBRA …………………….……..8 |  |
|  | HORA DO FIM DA ENTREVISTA   1. ***AGRADEÇA A RESPONDENTE E OFEREÇA-LHE O NOSSO PRESENTE*** 2. ***A SEGUIR COMPLETE AS QUESTÕES Q107-110*** | | HORAS……………………………………………\|___\|___\|  MINUTOS…………………………………….. \|___\|___\| |  |
