# Supplementary material for: Measuring Coverage in MNCH: Testing the Validity of Women's Self-Report of Key Maternal and Newborn Health Interventions during the Peripartum Period in Mozambique
Source: PLoS One. 2013 May 7;8(5):e60694. doi: 10.1371/journal.pone.0060694 (PMC3646219; doi:10.1371/journal.pone.0060694)
Supplement: Checklist S2 — English version of the questionnaire. (DOCX) [file pone.0060694.s002.docx]

**WOMAN’S MATERNAL RECALL QUESTIONNAIRE**

| **#** | **Question** | **Response codes** | **SKIP TO** |
| --- | --- | --- | --- |
| 1. **COVER SHEET INFORMATION** | | | |
|  | INTERVIEWER NAME I (Select) | \|___\|___\|___\| |  |
|  | FACILITY Name | \|___\|___\|___\| |  |
|  | CLIENT’S Name **TO BE RECORDED BY THE INTERVIEWER PRIOR TO INTERVIEW** | \|____\| |  |
|  |  |  |  |
|  |  |  |  |
|  |  |  |  |
|  | URBAN/RURAL RESIDENCE | URBAN………………………………………………..………1  RURAL………………………………………………………….2 |  |
|  | DATE OF HOUSEHOLD VISIT #1 | \|  \|  \|  \|  \|  \|  \| \| --- \| --- \| --- \| --- \| --- \| --- \|   D D M M Y Y |  |
|  | OUTCOME OF HOUSEHOLD VISIT #1  **COMPLETE AT END OF INTERVIEW OR END OF ATTEMPT TO CONTACT** | STARTED INTERVIEW……………………..……….5  LOCATED, BUT HAD TO  RESCHEDULE…………………………………………………2  REFUSED INTERVIEW………………………….………..3  COULD NOT RELOCATE……………………….………..4  WOMAN WAS INCAPACITATED………………..….6  PASSED AWAY………………………………………………7 | END  END  END  END  END  END |
|  | DATE OF HOUSEHOLD VISIT #2 | \|  \|  \|  \|  \|  \|  \| \| --- \| --- \| --- \| --- \| --- \| --- \|   D D M M Y Y |  |
|  | OUTCOME OF HOUSEHOLD VISIT #2  **COMPLETE AT END OF INTERVIEW OR END OF ATTEMPT TO CONTACT** | Continued D INTERVIEW…………………….……….1  PARTIALLY COMPLETE INTERVIEW………………5  REFUSED INTERVIEW…………………………………..3  WOMAN WAS INCWoman_Select%>APACITATED……………….….6 | END  END  END  END |
|  | TIME INTERVIEW BEGINS: USE THE 24 HOUR CLOCK | HOURS……………………………….\|___\|___\|  MINUTES………………………….. \|___\|___\| |  |
|  | WHERE IS THE INTERVIEW TAKING PLACE? | WOMAN’S HOME…………………………………………1  OTHER’S HOME…………………………………………….2  OTHER – SPECIFY 3 |  |
|  | RECORD THE LATITUDE AND LONGITUDE TAKEN AT THE PLACE OF INTERVIEW | LAT   \|  \|  \|  \| \| --- \| --- \| --- \|   LAT  LONG   \|  \|  \|  \| \| --- \| --- \| --- \| |  |
|  | Olá. Chamo-me _________________. Sou um entrevistador representando o Ministério de Saúde e o Programa  Integrado de Saúde Materna e Infantil patrocinado por USAID. Estamos realizando um estudo de mulheres que  deram à luz recentemente em Moçambique, para entender melhor o sua experiência de dar a luz. Se você concorda,  eu gostaria de entrevistar-lhe sobre a sua experiencia dando a luz. A entrevista vai durar mais ou menos 30 minutos.  Por favor, saiba que o decisão para participar nesta entrevista é completamente voluntária, e pode desistir em  qualquer altura. Podia sentir algum incómodo durante a entrevista por causa da natureza das perguntas. Podia haver  nenhum benefício directo para você por estar neste estudo unidade sanitária, mais os resultados vão informar os  actividades neste unidade sanitária. Nem o seu nome nem a data dos serviços será fornecido em qualquer dado  partilhado, portanto a sua identidade e qualquer informação sobre você permanecerá completamente confidencial.   Pode telefonar para o investigador principal a Jim Ricca a +258-82-305-3916 caso tenha alguma dúvida ou  preocupação relativamente a este estudo.   Se tiver qualquer pergunta sobre os seus direitos como um participante neste estudo, ou se pensa que não  foi tratada numa forma justa, pode ligar para Dr Francisco Mbofana no Comite Nacional de Bioetica a  +258-82-328-9550.  Uma pequena oferta vai ser fornecido a você em agradecimento para o seu tempo, se você concorda em ser  entrevistada ou não, A small gift of [NAME OF GIFT] will be provided to you today as thanks for your time,  whether you agree to be interviewed or not.  Tem alguma pergunta para mim? Tenho a sua permissão para continuar?  \|___\| SIM \|___\| NAO  Assinatura do entrevistador:  (Indica a disponibilidade da inquirida em participar  Olá. Chamo-me _________________. Sou um entrevistador representando o | |  |

Add consent checkbox

| **2. BACKGROUND INFORMATION** | | | |
| --- | --- | --- | --- |
|  | How old were you at your last birthday?  PROBE IF DK, OTHERWISE ESTIMATE AN AGE FOR THE WOMAN | AGE IN YEARS \|___\|___\| |  |
|  | Did you ever attend school? | YES……………………….………………………………………1  NO…………………………………….…………………………0 | 204 |
|  | How many years of school have you completed? | NONE..........................................................................00  PRIMÁRIO EP1............................................................01  PRIMÁRIO EP2............................................................02  SECONDARY ESG1.....................................................03  SECONDARY ESG2......................................................04  ELEMENTARY TECHNICAL...........................................05  BACIC TECHNICAL.....................................................06  INTERMEDIATE TECHNICAL.......................................07  TEACHING SCHOOL …………........................................08  UNIVERSITY OR ABOVE..............................................09 |  |
|  | Can you read a newspaper or letter, easily, with difficulty or not all? | EASILY…………………………..………..……………………..1  WITH DIFFICULTY………………………….……………….2  NOT AT ALL…………………………………..……………….3 |  |
|  | Are you now single, married, living together, divorced, separated or widowed? | SINGLE………………………………………………………….1  MARRIED………………………………………………………2  LIVING TOGETHER……………………..………………..3  SEPARATED………………………………………………….4  WIDOWED……………………………………………………5 | 208  208  208 |
|  | Has your husband ever attended school? | YES…………………………………………….…………………..1  NO……………………………………………..………………….0  DON’T KNOW…………………………….………………….8 | 208 |
|  | How many years of school has your husband completed? | NONE..........................................................................00  PRIMÁRIO EP1............................................................01  PRIMÁRIO EP2............................................................02  SECONDARY ESG1.....................................................03  SECONDARY ESG2......................................................04  ELEMENTARY TECHNICAL...........................................05  BACIC TECHNICAL.....................................................06  INTERMEDIATE TECHNICAL.......................................07  TEACHING SCHOOL …………........................................08  UNIVERSITY OR ABOVE..............................................09 |  |
|  | What is your main occupation? That is, what kind of work do you mainly do? | MAID.....................................................................00  STUDENT................................................................01  FARMING................................................................02  PUBLIC SECTOR EMPLOYEE ...................................03  SELF EMPLOYED......................................................04  PRIVATE SECTOR EMPLOYEE..................................05  OTHER___________________________________08  (SPECIFY) | 211 |
|  | Do you work outside the home? | YES………………………………………………………….……..1  NO…………………………………………………………..…….0 |  |
|  | Do you work for someone in your family, someone else or are you self-employed? | WORK FOR FAMILY MEMBER………………………..1  WORK FOR SOMEONE ELSE………………..…………2  SELF-EMPLOYED………………………………..………….3 |  |
|  | Do you have:  A source of tapped water inside your house?  Electricity in your house?  A cell phone? | YES NO   1. 0   1 0  1 0 |  |
|  | Do you own your house or do you rent? | OWN HOUSE………………………………………………..1  RENT HOUSE………………………………………………..2  OTHER (SPECIFY)………………………………………….8  ____________________________________ |  |

| **3. REPRODUCTION** | | | | |
| --- | --- | --- | --- | --- |
|  | Have you been pregnant since your delivery in Sept/October 2011? | YES………………………………………………..……………….1  NO……………………………………….………………………..0 | | 303 |
|  | What was the outcome of this most recent pregnancy (since Sept/Oct 2011)? Was it a miscarriage, induced abortion, stillbirth (that is, a baby who was born dead) or live birth? | MISCARRIAGE……………………………………..………..1  INDUCED ABORTION……………………………………..2  STILLBIRTH………………………………………….………….3  LIVE BIRTH…………………………………………….………..4  STILL PREGNANT……………………………………………5 | |  |
|  | How many children to whom you have given birth live with you now? | NUMBER OF CHILDREN LIVING WITH  WOMAN  \|____\|____\| | |  |
|  | How many children to whom you have given birth are not living with you now? | NUMBER OF CHILDREN NOT LIVING WITH  WOMAN  \|____\|____\| | |  |
|  | How many children to whom you have given birth were born alive but later died? | NUMBER OF CHILDREN BORN ALIVE BUT LATER DIED  \|____\|____\| | |  |
|  | How many stillbirths have you ever had, that is a baby who was born dead? | NUMBER OF STILLBIRTHS  \|____\|____\| | |  |
|  | How many times have you been pregnant, but lost the pregnancy before delivery? | NUMBER OF PREGNANCY LOSSES BEFORE DELIVERY  \|____\|____\| | |  |
|  | So, the total number of times that you have been pregnant is (INSERT TOTAL FOR QUESTIONS 303-307). | TOTAL PREGNANCIES  \|____\|____\| | |  |
|  | 308A. Is this correct? IF NOT CORRECT, REVIEW QUESTIONS 303-307, FIND MISTAKE AND RECORD CORRECT ANSWER IN 308, THEN CIRCLE 1 YES. IF NOT CORRECT DUE TO TWINS/MULTIPLE BIRTH, LEAVE RESPONSE AS IS AND CIRCLE 1 – YES CORRECT. | YES | |  |
|  | Now I have some questions about your delivery in Sept/Oct 2011. **EMPHASIZE TO RESPONDENTS WHO HAVE HAD A PREGNANCY OUTCOME SINCE SEPT/OCT 2011 THAT THESE QUESTIONS ARE ABOUT THE PREGNANCY THAT ENDED IN SEPT/OCT AND NOT HER MOST RECENT PREGNANCY.** | | |  |
|  | Was your delivery in Sept/Oct 2011 a single birth or a multiple birth (twins)? | | SINGLETON…………………………………….…………….1  MULTIPLE………………………………………….………….2 |  |
|  |  |  |  |  |
|  | What was the outcome for your delivery in Sept/Oct 2011? Was your baby/ies born alive or stillborn? **IF WOMAN DELIVERED TWINS, RECORD ANSWER FOR 2^ND^ TWIN ONLY** | | BORN ALIVE………………………………………………..1 STILLBORN………………………………………..………..2 | 315 |
|  | Do you know, did your baby die before your labor pains started for your delivery in Sept/Oct 2011? | | YES……………………………..………………………………...1  NO…………………………………………………………………0  DON’T KNOW……………..………………………………..8 |  |
|  | Were you shown your baby who was born in Sept/Oct 2011? | | YES………………………………………….………………………1  NO………………………………………….………………………0  DON’T KNOW………………………..……………………….8 |  |
|  | Was your baby’s skin intact or was your baby’s skin peeling? | | SKIN INTACT………………………..……………………….1  SKIN PEELING……………………….………………………2  DON’T KNOW………………………..……………………..8 |  |
|  | 315A Was a name given to the baby  315B What was the name given to your baby? | | Yes … 1 No ….. 0  BABY NAME_________________________ | 316 |
|  | Is **[NAME]** still alive? | | YES……………………………………………………..……..1  NO…………………………………………..………………..0 | 318 |
|  | How old was **[NAME]** (when he/she died?  RESPOND IN DAYS IF DEATH BEFORE ONE MONTH; RESPOND IN MONTHS IF DEATH AT AGE 1+ MONTHS | | DAYS ..............................\|__\|__\|    MONTHS....................... \|__\| __\| |  |
|  | Is/was **[NAME]** a boy or a girl? | | BOY………………………………………………………………..1  GIRL……………………………………………………………….2 |  |
|  | Do you have a health card with information about yourself? | | YES……………………………………………………..…………..1  NO…………………………………………………………………..0 | 321 |
|  | Can I see your health card? RESPOND YES IF WOMAN SHOWS YOU HER CARD | | YES……………………………………………………………..……1  NO……………………………………………………..……………0 |  |
|  | Do you have a health card for [**NAME]**? | | YES…………………………………………..…………………….1  NO…………………………………………..…………………….0 | 401 |
|  | Can I see **[NAME’S]** card? | | YES………………………………………………..……………….1  NO…………………………………………………………..…….0 |  |

| **4. HEALTH CARE FOR THE DELIVERY IN SEPT/OCT 2011** | | | | |
| --- | --- | --- | --- | --- |
| Now I have some questions about health care you have received. I will start with a question about your previous pregnancies and then I will ask you a number of questions about the care you received during your pregnancy and delivery in Sept/Oct 2011. We understand that some of these questions may be difficult for women to answer. So, if you just do not know the answer or cannot remember the answer, just tell me. | | | | |
|  | Have you ever had a birth by cesarean delivery? That is, a delivery where they cut your belly open to deliver the baby/ies? | YES…………………………………………………………………..1  NO…………………………………………………………………..0  DON’T KNOW/CAN’T REMEMBER…………………..8 | |  |
|  | Did you see anyone for antenatal care for the pregnancy that ended in Sept/Oct 2011? | YES……………………………………………………………1  NO…………………………………………………………...0 | | 406 |
|  | Whom did you see for antenatal care for the pregnancy that ended in Sept/Oct 2011?  LIST ALL PROVIDERS VISITED | YES NO   1. DOCTOR ……………….......... 2. NURSE ……………..………….…   D. TBA………..……………………....  E.OTHER COMMUNITY VOL.. | |  |
|  | Where did you receive antenatal care for your pregnancy that ended in Sept/Oct 2011?  LIST MULTIPLE LOCATIONS IF NECESSARY | PUBLIC: YES NO  A. GOVT HOSPITAL………………………….1 2  B.GOVTHEALTH CENTER….……………. 1 2  C.GOVT HEALTH POST…………………….1 2  D.OTHER PUBLIC SECTOR ……………….1 2  (SPECIFY)  ______________________________________  PRIVATE:  E.PRIVATE HOSPITAL ………..............1 2  F.PRIVATE HEALTH CENTER……………1 2  G.OTHER PRIVATE SECTOR……………..1 2  (SPECIFY)  ____________________________________  NONGOVERNMENTAL (NGO):  H.NGO HEALTH FACILITY…………………1 2 | |  |
|  | How many times did you receive antenatal care for the pregnancy that ended in Sept/Oct 2011? (Enter 8 for DK/Can’t remember) | NUMBER OF ANC VISITS  \| __\|  DON’T KNOW/DON’T REMEMBER……………..........08 | |  |
|  | Where did you go to deliver the pregnancy that ended in Sept/Oct 2011?  If not able to identify public, private, or NGO, write down name of the facility:  _________________________ | PUBLIC: YES NO  A.GOVT HOSPITAL………………………….1 2  B.GOVTHEALTH CENTER….……………. 1 2  C.GOVT HEALTH POST…………………….1 2  D.OTHER PUBLIC SECTOR ……………….1 2  (SPECIFY)  ______________________________________  PRIVATE:  E.PRIVATE HOSPITAL ………..............1 2  F.PRIVATE HEALTH CENTER……………1 2  G.OTHER PRIVATE SECTOR……………..1 2  (SPECIFY)  ____________________________________  NONGOVERNMENTAL (NGO):  H.NGO HEALTH FACILITY……………… 1 2 | |  |
|  | After you arrived at the health facility for the birth of [NAME(S)] in Sept/Oct 2011, did someone take your blood pressure? | YES……………………………………..……………………………1  NO………………………………………………………..…………0  DON’T KNOW/CAN’T REMEMBER……………..……8 | |  |
|  | Did someone ask you to give them a urine sample? | YES………………………………………………………….………1  NO………………………………………………………….………0  DON’T KNOW/CAN’T REMEMBER…………….……8 | |  |
|  | Now I have some questions about HIV testing. However, please know in advance that we will not ask you your HIV status.  While you were at the health facility for the birth of [NAME(S)] in Sept/Oct 2011, did someone ask you what your HIV status was? | YES…………………………………………………………………1  NO…………………………………………………………………0  DON’T KNOW/CAN’T REMEMBER…………………8 | |  |
|  | While you were at the health facility for the birth of [NAME(S)] in Sept/Oct 2011, did someone offer you an HIV test? | YES……………………………………………………………..……1  NO……………………………………………………………………0  DON’T KNOW/CAN’T REMEMBER…………………… 8 | |  |
|  | While you were at the health facility for the birth of [NAME(S)] in Sept/Oct 2011, did anyone test you for HIV? | YES……………………………………………………………………1  NO…………………………………………………………..……… 0  DON’T KNOW/CAN’T REMEMBER……………..……. 8 | |  |
|  | For your birth of [NAME(S)] in Sept/Oct 2011, did your labor start at home, en route to the health facility, once you were already at the hospital or did you never go into labor? | HOME………………………………………………………..………1  EN ROUTE TO FACILITY…….………………………………………………………2  ONCE AT FACILITY…………………………………….……….3  NEVER WENT INTO LABOR …………………………..…..4  DON’T KNOW/CAN’T REMEMBER……….………..….8 | |  |
|  | For your birth of [NAME/(S)] in Sept/Oct 2011, did your labor start spontaneously or did someone do something to bring on your labor? | SPONTANEOUS LABOR…………………………………….1  SOMEONE DID SOMETHING TO START  LABOR………………………………………………………………2  DON’T KNOW/CAN’T REMEMBER…………………… 8 | | 415  415 |
|  | For your birth of [NAME/S] in Sept/Oct 2011, what was done to bring on your labor? | IV LINE INSERTED IN ARM……………………..…………..1  OTHER (SPECIFY)………………………………………………..8  _____________________________________  _____________________________________ | |  |
|  | For your birth in Sept/Oct 2011, did anyone offer you the opportunity to have a companion with you during your labor and delivery? | YES……………………………………..……………………………1  NO……………………………………………………………………0  DON’T KNOW/CAN’T REMEMBER…………..………8 | |  |
|  | For your delivery in Sept/Oct 2011, did you choose to have a companion with you during labor/delivery? | YES…………………………………………………………..………1  NO……………………………………………………………………0  DON’T KNOW/CAN’T REMEMBER……………………8 | | 418  418 |
|  | For your delivery in Sept/Oct 2011, who did you choose to accompany you during labor OR delivery of [NAME(S)]? | MOTHER………………………………………………………….1  MOTHER-IN-LAW……………………………..…………….2  SISTER……………………………………………………………..3  OTHER FAMILY MEMBER………………….…………….4  SISTER IN LAW………………………………………..……………5  TRADITIONAL BIRTH ATTENDANT……………………6  OTHER (SPECIFY)……………………………………………..8  ______________________________________ | |  |
|  | For your delivery in Sept/Oct 2011, did anyone ask you if you wanted to get up and walk around while you were in labor? | YES……………………………………………………….……………1  NO………………………………………………………….…………0  DON’T KNOW/CAN’T REMEMBER………….…………8 | |  |
|  | For your delivery in Sept/Oct 2011, did you choose to get up and walk around during labor? | YES………………………………………………………….…………1  NO……………………………………………………….……………0  DON’T KNOW/CAN’T REMEMBER………….…………8 | |  |
|  | For your delivery in Sept/Oct 2011, did anyone ask you if you wanted to drink liquids or eat any food while you were in labor? | YES……………………………………………………………………1  NO……………………………………………………………………0  DON’T KNOW/CAN’T REMEMBER……………………8 | |  |
|  | For your delivery in Sept/Oct 2011, did you choose to drink liquids or eat food during labor? | YES……………………………………………………………………1  NO……………………………………………………………………0  DON’T KNOW/CAN’T REMEMBER…………..……… 8 | |  |
|  | For your delivery in Sept/Oct 2011, did you have privacy while you were in labor, that is, were you surrounded by some curtains? | YES…………………………………………………………..………1  NO…………………………………………………………..………0  DON’T KNOW/CAN’T REMEMBER……………………8 | |  |
|  | For your delivery in Sept/Oct 2011, was anything done to speed up or to strengthen your labor? | YES……………………………………………………………………1  NO…………………………………………………….………………0  DON’T KNOW/CAN’T REMEMBER………………….…8 | | 425  425 |
|  | For your delivery in Sept/Oct 2011, what was done to speed up or to strengthen your labor? | RECEIVED AN INJECTION DURING LABOR…………1  GIVEN MEDICATION IN AN IV LINE DURING LABOR……………………………………………………..……...2  OTHER (SPECIFY)………………………………………………8  ________________________________________ | | 426 |
|  | For your delivery in Sept/Oct 2011, did you receive any injections during labor, that is *before the birth of your baby/ies*? | YES……………………………………………………………..………1  NO……………………………………………………………..………0  DON’T KNOW/CAN’T REMEMBER…………..…………8 | |  |
|  | For your delivery in Sept/Oct 2011, during your labor, were you covered with a drape, or was your body exposed to people around you? | YES, COVERED WITH DRAPE…………………..………….1  NOT COVERED..……………………………………..………….0  DON’T KNOW/CAN’T REMEMBER……………..………8 | |  |
|  | For your delivery in Sept/Oct 2011, how was the baby lying in your womb *right before birth*? Was [NAME’S] head down, feet down or was [NAME(S)] lying sideways across your womb? IF TWINS, REFER TO THE 2^ND^ TWIN | HEAD DOWN………………………………………….…………..1  FEET DOWN……………………………………………………….2  SIDEWAYS/ACROSS WOMB……………………………….3  DON’T KNOW/CAN’T REMEMBER……………………..8 | |  |
|  | For your delivery in Sept/Oct 2011, did a health care provider ask you what position you wanted to choose during your labor OR for the delivery of your baby? | YES………………………………………………………….…………1  NO…………………………………………………………….………0  DON’T KNOW/CAN’T REMEMBER…………….………8 | |  |
|  | For your delivery in Sept/Oct 2011, who assisted you with the birth of [NAME(S)]?  LIST ALL PROVIDERS PRESENT | YES NO  DOCTOR ………………..........1 2  NURSE ……………..………….…1 2  TBA………..…………………….....1 2  OTHER……………………………...1 2  (SPECIFY)  _______________________________________ | |  |
|  | Was [NAME] delivered by cesarean, that is, did they cut your belly open to take the baby out? | YES………………………………………………………..……………1  NO………………………………………………………………………0  DON’T KNOW/CAN’T REMEMBER………………………8 | | 433  433 |
|  | Did you have your cesarean operation before you went into labor or after your labor had already started? | BEFORE LABOR STARTED…………………………….1  AFTER LABOR STARTED……………………………….2  DON’T KNOW, CAN’T REMEMBER……………….8 | |  |
|  | What was the reason for your cesarean operation? | The doctor/nurse told me I had to. ………………..01  I was bleeding…………………………………………..…...02  The baby was stuck…………………………………..….…03  I was in labor pain for a long time……………..……04  The baby was not in the right position……..…...05  I had a disease……………………………………………....06  My womb was broken/ruptured………………......07  There were problems with the baby……………...08  There was no medical reason…………………….…..10  Don’t know….……………………………………………..….00 | | ALL GO TO 435 |
|  | Was [NAME] delivered using forceps ( that is, an instrument to help pull the baby out) or suction to help pull the baby out? IF TWINS, REFER TO 2^ND^ TWIN | YES FORCEPS……………………………………………..………1  YES SUCTION………………………………………………..……2  NO………………………………………………………………..……0  DON’T KNOW/CAN’T REMEMBER………………..……8 | |  |
|  | For your delivery in Sept/Oct 2011, what position were you in when you actually delivered [NAME(S)]? That is, were you on your back, on your hands and knees, squatting or in another position? | ON YOUR BACK………………………………………..………..1  ON HANDS AND KNEES………………………………………2  SQUATTING……………………………………………..……….3  OTHER (SPECIFY)  _______________________________________8 | |  |
|  | For your delivery in Sept/Oct 2011, shortly before you delivered your baby, did anyone cut the opening of your vagina (episiotomy) to make more room for the baby’s head? | YES………………………………………………………………………1  NO………………………………………………………………………0  DON’T KNOW/CAN’T REMEMBER………………………8 | |  |
|  | **CHECK RESPONSE TO 430. IF BABY DELIVERED BY CESAREAN SECTION, GO TO QUESTION 449.** | | |  |
|  | For your delivery in Sept/Oct 2011, as it came close to the time you delivered your baby and you were pushing/bearing down, did anyone stand above you and push downward/apply pressure on your womb? | YES……………………………………………………………………..1  NO……………………………………………………………………..0  DON’T KNOW/CAN’T REMEMBER………………..……8 | |  |
|  | For your delivery in Sept/Oct 2011, when [NAME/S] was/were born, what body part came out first? [NAME’S] head, feet (breech) or another body part? IF TWINS, REFER TO 2^ND^ TWIN | HEAD FIRST…………………………………………………………1  FEET FIRST/BREECH……………………………………………2  ARM/LEG CAME OUT FIRST ………………………..….…3  DON’T KNOW/CAN’T REMEMBER……………………..8 | |  |
|  | Now I have some questions about you and your baby shortly after she/he was born (in Sept/Oct 2011). (IF TWINS, REFER TO 2^ND^ TWIN) | | |  |
|  | For your delivery in Sept/Oct 2011: Now, I have some questions about the time *just after the delivery of [NAME(S).]* In the first few minutes after the delivery of your baby did anyone give you an injection in your thigh or buttock? | | YES…………………………………………………………..………1  NO…………………………………………………………..………0  DON’T KNOW/CAN’T REMEMBER…………..………8 |  |
|  | For your delivery in Sept/Oct 2011: *Just after the delivery of [NAME(S).]* In the first few minutes after the delivery of your baby, did anyone give you medication intravenously (through a tube in your arm)? | | YES………………………………………………………………..…1  NO……………………………………………………………………0  DON’T KNOW/CAN’T REMEMBER……………..……8 |  |
|  | For your delivery in Sept/Oct 2011: Just after the delivery of [NAME(S).] In the first few minutes after the delivery of your baby did anyone give you tablets to swallow or hold in your mouth? | | YES…………………………………………………………….………1  NO…………………………………………………………………… 0  DON’T KNOW/CAN’T REMEMBER……………..……..8 |  |
|  | For your delivery in Sept/Oct 2011: Just after the delivery of [NAME(S).] In the first few minutes after the delivery of your baby did anyone place tablets in your rectum? | | YES…………………………………………………..………………1  NO…………………………………………………..………………0  DON’T KNOW/CAN’T REMEMBER…………..………8 |  |
|  | Can you remember if you received this [injection/medication] BEFORE the delivery of the placenta? | | YES……………………………………………………..……………1  NO………………………………………………………..…………0  PLACENTA DELIVERED RIGHT AFTER BABY……...3  DON’T KNOW/CAN’T REMEMBER………………..…8 |  |
|  | For your delivery] in Sept/Oct 2011, after the delivery of [NAME(S)], did the birth attendant help you deliver the placenta, that is, did he/she place his/her hand firmly on your lower abdomen with one hand and hold the umbilical cord in the other hand? | | YES……………………………..………………………………….…1  NO………………………………….…………………..……………0  PLACENTA DELIVERED IMMEDIATELY AFTER BIRTH WITHOUT ASSISTANCE……………………..….3  DON’T KNOW/CAN’T REMEMBER………….………..8 |  |
|  | After the delivery of [NAME(S)] (in Sept/Oct 2011), in the first few minutes *after the delivery of the placenta*, did anyone give you an injection in your thigh? | | YES……………………………………………………………………1  NO…………………………………………………..………………0  DON’T KNOW/CAN’T REMEMBER…………..………8 |  |
|  | After the delivery of [NAME(S)] (in Sept/Oct 2011), in the first few minutes *after the delivery placenta*, did anyone give you tablets to take or hold in your mouth? | | YES……………………………………………………………………1  NO…………………………………………………..………………0  DON’T KNOW/CAN’T REMEMBER…………..………8 |  |
|  | For your delivery in Sept/Oct 2011, after the delivery of your baby, did the birth attendant firmly massage your lower abdomen to help make your womb contract (become hard)? | | YES……………………………………………………………1  NO……………………………………………………………0  DON’T KNOW/CAN’T REMEMBER……………8 |  |
|  | **446A.** For your delivery in Sept/Oct 2011, after the delivery of the placenta, did the birth attendant firmly massage your lower abdomen to help make your womb contract? | | YES……………………………………………………………1  NO……………………………………………………………0  DON’T KNOW/CAN’T REMEMBER……………8 |  |
|  | When [NAME(S)] was born in Sept/Oct 2011, what instrument was used to cut the umbilical cord(s)? | | NEW RAZOR BLADE…………………………..….…………1  USED RAZOR BLADE………………………………………..2  SCISSORS………………………………….………….…………..3  THREAD…………………………………….……………………...4  OTHER (SPECIFY)……………………………………………….5  ________________________________________  DON’T KNOW/CAN’T REMEMBER…………………….8 |  |
|  | For your delivery in Sept/Oct 2011, did your birth attendant/s place his/her hand inside your womb after delivery of your baby? | | YES……………………………………………………………1  NO……………………………………………………………0  DON’T KNOW/CAN’T REMEMBER……………8 |  |
|  | Was your baby dried off with a towel immediately after his/her birth, within a few minutes of delivery? IF TWINS, REFER TO 2^ND^ TWIN | | YES……………………………………………………………1  NO……………………………………………………………0  DON’T KNOW/CAN’T REMEMBER……………8 |  |
|  | Did someone place the baby on your chest, against your skin, immediately after delivery of the baby?  IF TWINS, REFER TO 2^ND^ TWIN | | YES……………………………………………………………1  NO……………………………………………………………0  DON’T KNOW/CAN’T REMEMBER……………8 | 453  453 |
|  | Was your baby wrapped in a cloth while lying against your chest or was your baby naked against your skin? | | WRAPPED IN CLOTH…………………………………………1  BABY NAKED AGAINST YOUR SKIN..…………………2  DON’T KNOW/CAN’T REMEMBER……………………8 | 454  454 |
|  | (If naked against skin), was your baby covered with a towel or cloth while lying against your skin? | | YES……………………………………………………………1  NO……………………………………………………………0  DON’T KNOW/CAN’T REMEMBER……………8 | 454  454  454 |
|  | Was your baby wrapped in a towel or cloth immediately after birth? | | YES……………………………………………………………1  NO……………………………………………………………0  DON’T KNOW/CAN’T REMEMBER……………8 |  |
|  | Once your baby was born, did any of your birth attendants hold the baby upside down?  IF TWINS, REFER TO 2^ND^ TWIN | | YES…………………………………………………………..……1  NO…………………………………………………………..……0  DON’T KNOW/CAN’T REMEMBER…………..……8 |  |
|  | Once your baby was born, did any of your birth attendants slap your baby lightly to get him/her to cry? IF TWINS, REFER TO 2^ND^ TWIN | | YES…………………………………………………………..……1  NO…………………………………………………………..……0  DON’T KNOW/CAN’T REMEMBER…………..……8 |  |
|  | Did anyone weigh [NAME] just after birth? IF TWINS, REFER TO 2^ND^ TWIN | | YES…………………………………………………………………1  NO…………………………………………………………………0  DON’T KNOW/CAN’T REMEMBER…………………8 | 459  459 |
|  | What was [NAME’S] weight?  IF TWINS, REFER TO 2^ND^ TWIN | | WEIGHT IN GRAMS: \|___\|___\|____\|____\| |  |
|  | **INDICATE WHERE THE RESPONSE FOR WEIGHT CAME FROM** | | WEIGHT TAKEN FROM BABY HEALTH CARD…1  WEIGHT GIVEN BY MOTHER’S RESPONSE……2 |  |
|  | For your delivery in Sept/Oct 2011, was [NAME] born in the normal period of time, preterm or late? | | TERM (ON TIME)……………………………………………1  PRETERM (EARLY)..………………………………………..2  LATE ……………………………………………………………….3  DON’T KNOW/CAN’T REMEMBER……………..…..8 |  |
|  | For your delivery in Sept/Oct 2011, do you know how many weeks you were pregnant when you delivered [NAME]? | | WEEKS \|___\|___\|  DON’T KNOW, CAN’T REMEMBER……..00 |  |
|  | **CHECK QUESTIONS 310 AND 311. IF OUTCOME IS STILLBIRTH, GO TO Q463** | | |  |
|  | Did you breastfeed [NAME]? IF TWINS, REFER TO 2^ND^ TWIN | | YES……………………………………………………………1  NO……………………………………………………………0 | 463 |
|  | Did you breastfeed [NAME] within the first hour after delivery? IF TWINS, REFER TO 2^ND^ TWIN | | YES……………………………………………………………1  NO……………………………………………………………0  DON’T KNOW/CAN’T REMEMBER……………8 |  |
|  | Did you or anyone else give anything to the baby to eat or drink within the first hour after delivery? IF TWINS, REFER TO 2^ND^ TWIN | | YES……………………………………………………………1  NO……………………………………………………………0  DON’T KNOW/CAN’T REMEMBER……………8 |  |
|  | About how long after birth was your baby bathed for the first time? IF TWINS, REFER TO 2^ND^ TWIN | | WITHIN 1 HOUR…………………………….…………1  1-5 HOURS……………………………………………….2  6-24 HOURS……………………………………………..3  2-3 DAYS…………………………………………………..4  AFTER 3 DAYS…………………………………………..5  DON’T KNOW/CAN’T REMEMBER…………...8 |  |
|  | How many nights did you sleep in the health facility after the delivery of your baby? | | NIGHTS SLEPT AT  HEALTH FACILITY \|___\|  DON’T KNOW/CAN’T REMEMBER…………………8 |  |
|  | Do you know the birth date of (NAME)? | | YES…………………………………………….……………………1  NO…………………………………………………………………0 |  |
|  | What was the date of the birth of [NAME]? IF TWINS, REFER TO 2^ND^ TWIN | | BABY   \|  \|  \|  \|  \|  \|  \| \| --- \| --- \| --- \| --- \| --- \| --- \|   D D M M Y Y |  |
|  | During your entire stay at the health facility, did anyone physically mistreat you? That is, did anyone hit you, slap you, physically threaten you or in any other way cause you physical harm?  PLEASE SELECT ALL THAT APPLY | | YES, HIT……………………………………………………………1  YES, SLAPPED………………………………………………….2  YES, OTHER (SPECIFY)  ______________________________________3 NO PHYSICAL MISTREATMENT……………………..…4  DON’T KNOW/CAN’T REMEMBER…………………..8 |  |
|  | During your entire stay at the health facility, did anyone verbally mistreat you? That is, did anyone threaten you verbally or shout at you?  PLEASE SELECT ALL THAT APPLY | | YES, THREATENED…………………………………………….1  YES, SHOUT……………………………………………………..2  YES, OTHER (SPECIFY)……………………………………..3  ______________________________________ NO VERBAL MISTREATMENT…………………………..4  DON’T KNOW/CAN’T REMEMBER…………………..0 |  |
|  | TIME INTERVIEW ENDS   1. **THANK THE RESPONDENT AND OFFER HER OUR GIFT** 2. **COMPLETE Q107-110** | | HOURS……………………………………………\|___\|___\|  MINUTES………………………………………. \|___\|___\| |  |
